# A randomized, double-blind placebo-control study assessing the protective efficacy of an odour-based ‘push–pull’ malaria vector control strategy in reducing human-vector contact

**DOI:** 10.1038/s41598-023-38463-5

**Published:** 2023-07-11

**Authors:** Ulrike Fillinger, Adrian Denz, Margaret M. Njoroge, Mohamed M. Tambwe, Willem Takken, Joop J. A. van Loon, Sarah J. Moore, Adam Saddler, Nakul Chitnis, Alexandra Hiscox

**Affiliations:** 1 International Centre of Insect Physiology and Ecology (Icipe), Human Health Theme, Nairobi, 00100 Kenya; 2 Department of Epidemiology and Public Health, Swiss Tropical and Public Health Institute, Kreuzstrasse 2, Allschwil, Switzerland; 3 University of Basel, Petersplatz 1, Basel, Switzerland; 4 Laboratory of Entomology, Wageningen University & Research, P.O. Box 16, 6700 AA Wageningen, The Netherlands; 5 Vector Control Product Testing Unit (VCPTU), Department of Environmental Health and Ecological Sciences, Ifakara Health Institute, P.O. Box 74, Bagamoyo, Tanzania; 6 The Nelson Mandela African Institution of Science and Technology (NM-AIST), Tengeru, P.O. Box 447, Arusha, Tanzania; 7 Telethon Kids Institute, Perth, Australia; 8 Arctech Innovation Ltd., The Cube, Londoneast-Uk Business and Technical Park, Yew Tree Avenue, Dagenham, RM10 7FN UK

**Keywords:** Malaria, Entomology

## Abstract

Novel malaria vector control strategies targeting the odour-orientation of mosquitoes during host-seeking, such as ‘attract-and-kill’ or ‘push-and-pull’, have been suggested as complementary tools to indoor residual spraying and long-lasting insecticidal nets. These would be particularly beneficial if they can target vectors in the peri-domestic space where people are unprotected by traditional interventions. A randomized double-blind placebo-control study was implemented in western Kenya to evaluate: a ‘push’ intervention (spatial repellent) using transfluthrin-treated fabric strips positioned at open eave gaps of houses; a ‘pull’ intervention placing an odour-baited mosquito trap at a 5 m distance from a house; the combined ‘push–pull’ package; and the control where houses contained all elements but without active ingredients. Treatments were rotated through 12 houses in a randomized-block design. Outdoor biting was estimated using human landing catches, and indoor mosquito densities using light-traps. None of the interventions provided any protection from outdoor biting malaria vectors. The ‘push’ reduced indoor vector densities dominated by *Anopheles funestus* by around two thirds. The ‘pull’ device did not add any benefit. In the light of the high *Anopheles arabiensis* biting densities outdoors in the study location, the search for efficient outdoor protection and effective pull components needs to continue.

## Introduction

Since the year 2000, gains in malaria control in sub-Saharan Africa have been largely attributed to widespread indoor vector control with long-lasting insecticidal nets (LLINs) and to a smaller extent with indoor-residual spraying (IRS)^1^. It is clear that the use of these interventions alone will not be sufficient to achieve elimination of the disease^2,3^. Malaria transmission occurring outside of the house and during the early evening and early morning hours when people are not protected by the indoor insecticidal interventions is a major challenge for the current control tools^4–6^. Additionally, increasing levels of physiological and behavioural resistance to insecticidal tools limit the degree to which we can rely on these methods to achieve elimination and maintain current gains^7^. Vector control tools using alternative modes of action, for example by manipulating the odour-orientation of mosquito vectors^8–10^, are urgently sought to expand the toolbox for malaria vector control and provide options for tailoring integrated interventions to local eco-epidemiological settings^11,12^.

The concept of ‘push–pull’ in which the behaviour of insect pests is manipulated to drive them away from a host that should be protected (‘push’) and lure them towards an alternative attractive host or trap (‘pull’) has been well demonstrated in agricultural systems^13^ and is gaining traction as a concept which may be applied to the control of human malaria vectors in rural sub-Saharan Africa^8,9,14–17^. Spatial repellents have been proposed as ‘push’ interventions for use around the house to provide protection within the peri-domestic environment, in and around the home, during the hours when people are not yet sleeping under their bednets^18^. An efficient strategy for the spatial repellent application should deter mosquitoes from entering the protected space and hence reduce the number of bites people would receive within the activity range of the repellent. Mosquito coils are the best known and most used tool in this category. They work by releasing the spatial repellent vapour through combustion; however, this requires an open flame starter, is a fire hazard during operation, produces smoke which poses respiratory-related health risks^19–21^ and the duration of use is limited to a burn time of 4–12 h per unit. Whilst protective under supervised trial conditions, their efficiency is dependent on continuous household availability and use^22^. There is a need for flameless, battery-free, cost-effective, passive delivery methods for spatial repellents that provides sustained and safe protection over extended time to increase adherence of use and ultimately further reduce malaria transmission.

Recent studies under semi-field conditions in Kenya and Tanzania have shown that the use of passively emanated transfluthrin spatial repellents from fabric strips suspended around open eave gaps of houses can reduce human landing rates of *Anopheles gambiae s.s.* and *Anopheles arabiensis*^8,10,15,23^. ‘Pushing’ mosquitoes away from the protected locations may, however, put others at elevated risk since mosquitoes might be diverted to seek out people without protection^24^. To complement the repellents, it has been proposed to add odour-baited mosquito traps to ‘pull’ vectors in search of an attractive host into the trap^8,25,26^. The impact of such a mass-trapping approach on malaria has been assessed in a recent randomized controlled field trial in western Kenya. This trial associated a significant reduction in the indoor abundance of the malaria vector *Anopheles funestus* with the ‘pull’ intervention, a Suna trap baited with a synthetic chemical (MB5) blend^27^. Ideally ‘push–pull’ interventions would work synergistically with the push providing protection against mosquito biting and the pull reducing mosquito populations thus providing wider community benefits.

The impact of the combination of the two strategies, on reducing human biting rates has, so far, mainly been evaluated under standardized semi-field conditions in large enclosures with insectary-reared *An. gambiae* and *An. arabiensis* mosquitoes^15^ and in a very small field experiment with low replication^28^. The results from these studies are inconsistent, with very variable predictions of the protective efficacy (relative reduction of biting) of the spatial repellents (20–80%) and a lack of clarity about the added value of the ‘pull’ components. There were also design issues, which affect the comparability of outcomes, including small sample size, use of tools not commercially available and not consistent between studies, and absence of blinding and appropriate controls (i.e., a fabric strip and fan-powered trap might affect mosquito catches even without active ingredients). To complement the current evidence base, it was important to evaluate the ‘push–pull’ system under field conditions where the mosquito-chemical interaction is playing out on a much larger and more variable scale.

We implemented a randomized, double-blind placebo-control study in a rural irrigated rice agroecosystem in western Kenya, to investigate to what degree a push–pull strategy can protect people from potentially infectious mosquito bites inside the house and in the immediate surrounding outside the house (peri-domestic space) under real life conditions. The ‘push’ was provided by transfluthrin-treated fabric strips around eave gaps, and the ‘pull’ was provided by a Suna-trap^29^ baited with a 6-compound chemical blend (MB5 and 2-butanone^8^ set up 5 m away from the house with the spatial repellent. This particular ‘push–pull’ system had already been evaluated in semi-field studies, with *An. arabiensis* colonised from local populations^8^.

In this study outdoor vector-human contact was measured with human landing catches (HLCs)^30^ where mosquitoes were collected by human volunteers nightly between 1800 and 2300 h as they attempted to bite. The timing and duration of HLCs was selected to correspond with the hours when people are typically not sleeping under a LLIN and are exposed to mosquito bites. Potential indoor biting was estimated based on nightly mosquito catches from 1800 to 0600 h with CDC light traps next to a bed where a person was sleeping at night under an insecticidal bednet. The general aim of this study was to investigate if a ‘push’-only, ‘pull’-only or combined ‘push–pull’ strategy, in addition to the existing frontline intervention with long-lasting insecticidal nets (LLINs) were associated with reductions in the number of mosquitoes attempting to bite outdoors and indoors when compared with a control setting that only had LLINs combined with placebo ‘push’ and ‘pull’ elements (fabric strips and fan-powered traps without active ingredients). The protective efficacy of the interventions against indoor and outdoor biting by all abundant mosquito species was estimated with a hierarchical, negative-binomial Bayesian model.

## Results

The four treatment arms were allocated in a randomised block design to 12 houses; hence there were three houses per treatment on any given night. The treatments were rotated through the houses so that each house received all four treatments to account for variations between houses. Mosquito sampling was done over four consecutive nights a week and treatments were rotated on a weekly basis. The study was implemented for 17 weeks during the rainy season between September and December 2018. In that time, a total of 33,290 primary malaria vectors in the *An. gambiae* and *An. funestus* species complexes were collected combined for outdoor human landing catches (706 house-nights) and indoor CDC light trap catches (718 house-nights).

**Figure 1:**
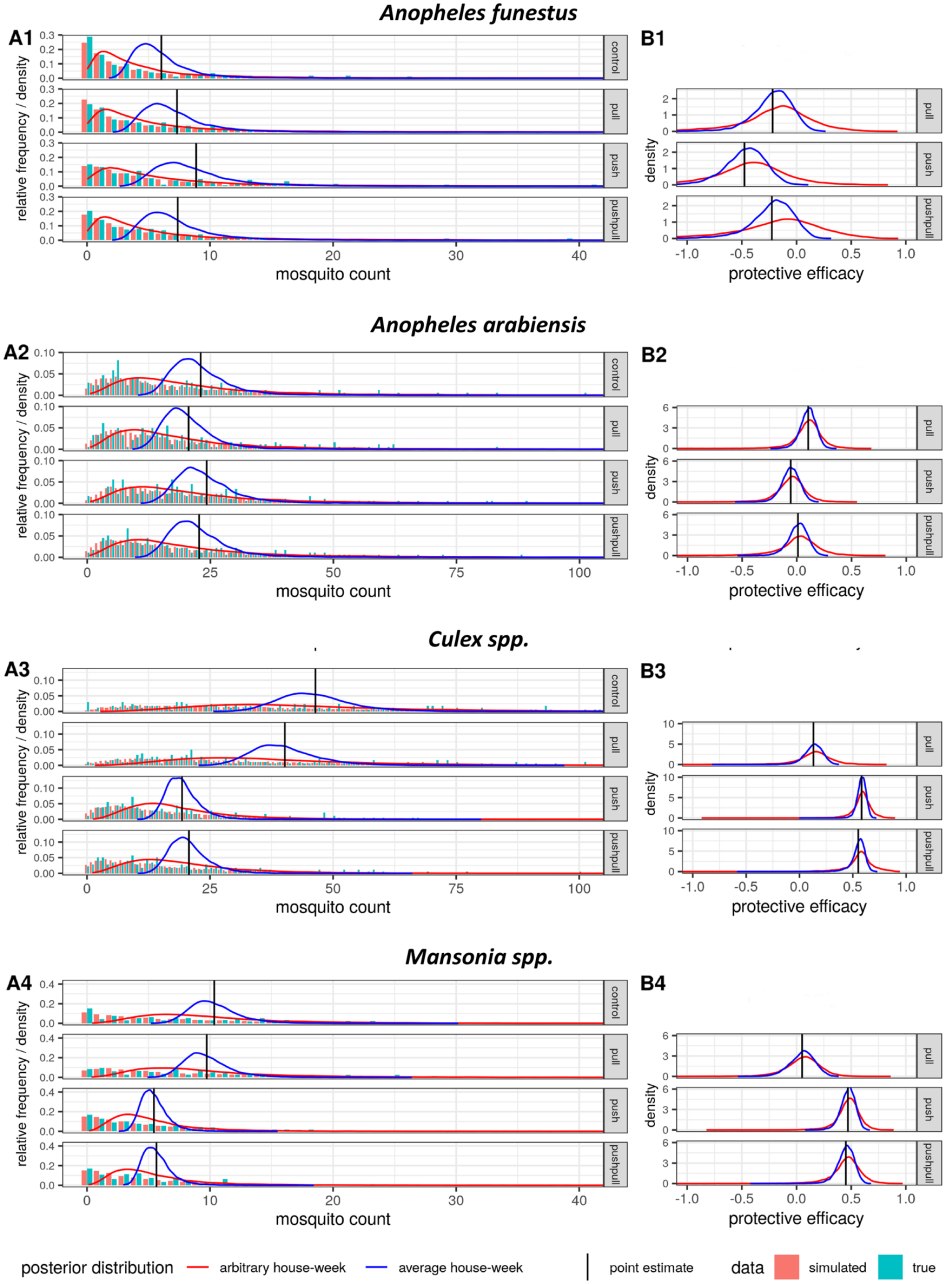
Outdoor mosquito densities and protective efficacies under the different interventions. (**A1–4**) shows the outdoor mosquito densities and (**B1–4**) the estimated protective efficacies for the mosquito species *An. funestus* (**1**), *An. arabiensis* (**2**), *Culex* (**3**) and *Mansonia* (**4**), under the different interventions. The red curves represent the estimated posterior distributions of the mean mosquito density in (**A1–4**) and of the protective efficacy in (**B1–4**) for an arbitrary house-week, and blue curves designate the same measures for an average house-week. The vertical black lines depict the point estimates (means of the posterior distribution) for the mean mosquito density in (**A1–4**) and for the protective efficacy in (**B1–4**). Note that the point estimates for an average and an arbitrary house-week are identical, i.e., corresponding red and blue curves always have the same mean (vertical black line). In (**A1–4**) the relative frequency bar plots show the real HLC data (turquoise) and simulated mosquito density data sampled from the fitted model (coral). The horizontal axis was truncated for presentation purposes and the following HLC data points were thus not plotted (but all were included in the inference): *An. arabiensis* control (107, 118); *Culex* control (106, 118, 131, 140, 156, 158, 275), ‘pull’ (105, 111, 118, 122, 124, 125, 137, 140, 155, 167, 180, 187, 271) and ‘push’ (126); *Mansonia* control (43, 47, 50, 54, 57) and ‘pull’ (46, 46, 47, 58). Note that the simulated data accounts for the intervention effect on both the mean and the dispersion parameter of the negative binomial model, and corresponds to an arbitrary house-week.

**Figure 2:**
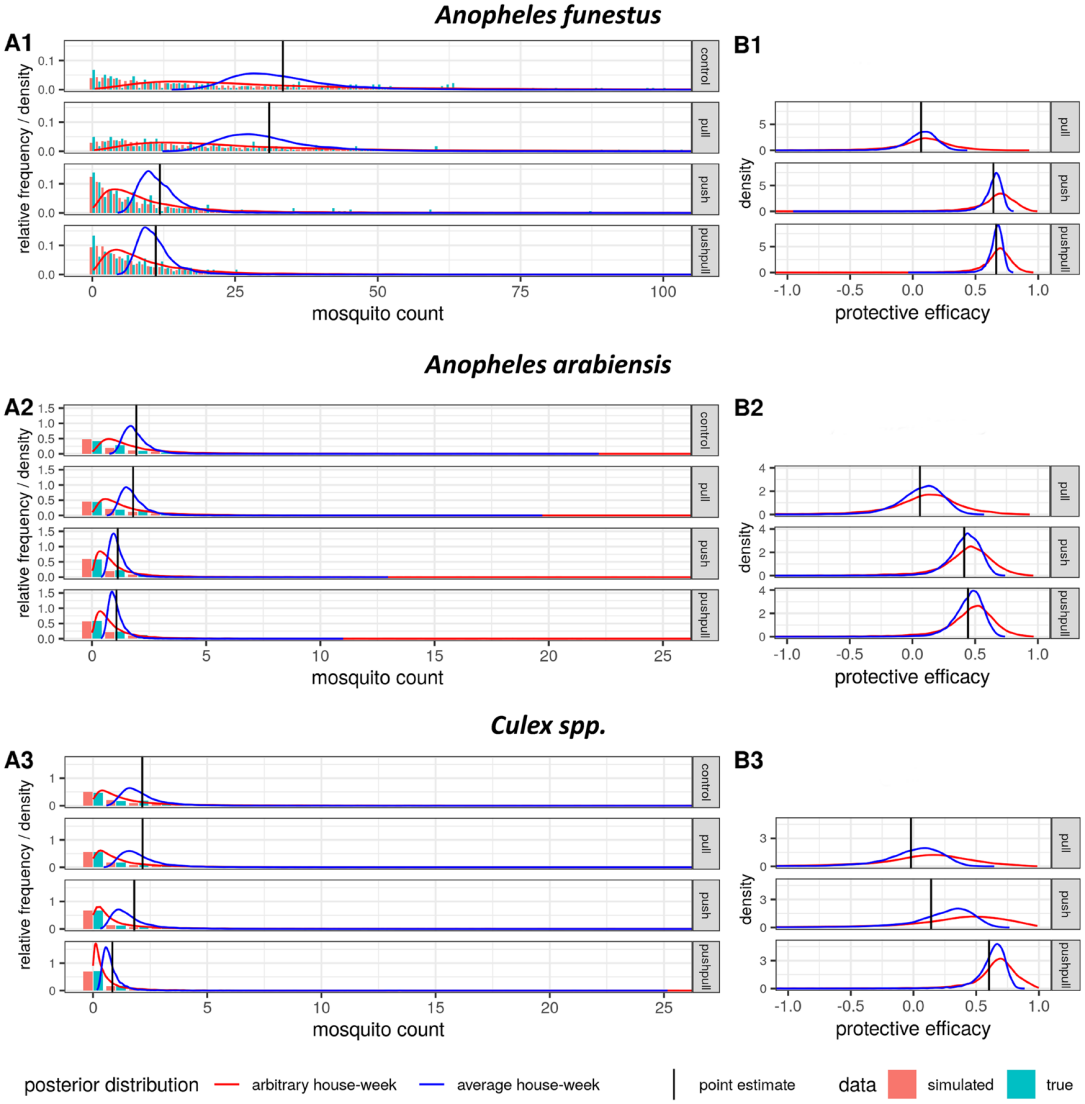
Indoor mosquito densities and protective efficacies under the different interventions. (**A1–3**) Shows the outdoor mosquito densities and (**B1–3**) the estimated protective efficacies for the mosquito species *An. funestus* (**1**), *An. arabiensis* (**2**) and *Culex* (**3**), under the different interventions. The red curves represent the estimated posterior distributions of the mean mosquito density in (**A1–3**) and of the protective efficacy in (**B1–3**) for an arbitrary house-week, and blue curves designate the same measures for an average house-week. The vertical black lines depict the point estimates (means of the posterior distribution) for the mean mosquito density in (**A1–3**) and of the protective efficacy in (**B1–3**). Note that the point estimates for an average and an arbitrary house-week are identical, i.e., corresponding red and blue curves always have the same mean (vertical black line). In (**A1–3**) the relative frequency bar plots show the real HLC data (turquoise) and simulated mosquito density data sampled from the fitted model (coral). The horizontal axis was truncated for presentation purposes and the following data points were thus not plotted (but they were included in the inference): *An. funestus ‘*pull’ (108, 118, 123, 179, 221); *An. arabiensis* control (44, 57) and ‘pull’ (37); *Culex* control (28), pull (49) and ‘push’ (27, 27).

The overall malaria vector density was similar indoors and outdoors, although the species composition was reversed in the two locations. Outdoors, using human landing catches (HLC), 13,640 *An. gambiae* s.l. were captured, while only 1294 specimens of this species were caught indoors by CDC light traps. Indoors, *An. funestus* s.l. predominated, with 14,556 specimens trapped in CDC light traps, while only 3800 were captured outdoors using HLC. Outdoor human landing catches also revealed a high density of the secondary malaria vector *An. coustani*, with a total of 2107 collected over 706 house-nights. Indoors, only 84 specimens of this species were trapped. Similarly, *Culex* and *Mansonia* species were highly abundant outdoor biting mosquitoes with 20,683 and 5427 specimens landing on human volunteers, respectively, whilst they were rarely encountered indoors (*Culex*: 978; *Mansonia*: 56). Figures 1 and 2, show the mosquito count data per species, and per treatment for indoor and outdoor samples. Supplementary Figs. S1–S4 show the same data per house and per week.

Almost all the *An. gambiae* s.l. (99%) specimens were molecularly amplified as *An. arabiensis* with only 1% amplifying as *An. gambiae* s.s. Similarly, *An. funestus* represented nearly exclusively (> 99%) the *An. funestus* complex whilst the remaining few amplified as *An. rivulorum* (< 1%). *Plasmodium* sporozoites were detected in 1% (52/5740) of all *An. arabiensis* and 2% (132/6180) of all *An. funestus* specimens. Percentages were similar for indoor and outdoor collections. These are conservative estimates, given that the sporozoite detection assays were done on pools of ten mosquitoes at a time.

All mosquito count data were analysed with a Bayesian negative binomial model, with separate inference for the outdoor (HLC), indoor (CDC light trap) and Suna trap catch data. Data points with missing information for one of the measurements, or missing information on the week and house of the measurement, were excluded from the analysis; these removed data points accounted for less than 5% of the data. A hierarchical, second level model structure (‘random effects’) was used to account for the dependence between data points collected from the same house or week. Thus, the model allowed for temporal and spatial heterogeneity in the intervention efficacy. Indoor and outdoor mosquito counts varied similarly across houses and weeks, but not proportionally to each other, as was shown by a less well-fitting alternative model that coupled indoor and outdoor bite counts per house or week (see Supplementary Methods). This indicates that not only the total abundance of mosquitoes varied across houses and weeks, but also their indoor vs. outdoor distribution.

For all estimates, uncertainty was quantified by Bayesian 95% highest density credible intervals (HDI), meaning values outside of the 95% HDI can be excluded with 95% probability. In order to ensure comparability with other studies, we follow the standard practice to quantify the uncertainty with respect to an average house and an average week. Hence, the reported interval estimates (HDI) are with respect to the first level model and do mask the variability of the outcome across different houses and weeks as modelled by the second level model. However, we believe that interval estimates should cover the full variability accounted for in the model that was used for the inference, ensuring a more robust intervention assessment. Thus, we report, in addition to the ‘average’ house-week analysis, interval estimates with respect to an ‘arbitrary’ house and an ‘arbitrary’ week in a separate section (‘arbitrary house-week analysis’), which contain all modelled variability.

### Primary outcome: reduction in host-seeking mosquito density

#### Outdoors

The biting density of primary malaria vectors in the outdoor area per house-night was estimated as 23.0 (95% HDI: 12.9 to 35.1) female *An. arabiensis* mosquitoes and 6.0 (95% HDI: 2.5 to 10.9) female *An. funestus mosquitoes* in the absence of any intervention (control; Table 1). The spatial repellent (‘push’) had no effect on the mean outdoor count for *An. arabiensis*, but, on the contrary, increased the risk of receiving bites outdoors from *An. funestus* (‘push’; Table 1)*.* Similarly, the ‘push–pull’ system had no effect on *An. arabiensis* densities and slightly increased the *An. funestus* density outdoors, while the latter estimate had less than 95% credibility. The odour-baited Suna trap (‘pull’) did neither affect the outdoor biting density of *An. arabiensis* nor *An. funestus*. Interestingly, the ‘push’ decreased the dispersion of the *An. funestus* and *An. arabiensis* outdoor counts and the ‘pull’ decreased the dispersion of the *An. arabiensis* outdoor count, meaning that the counts were less variable under the intervention as compared to the respective controls (see Supplementary Note).

**Table 1:** Mean outdoor and indoor mosquito densities as measured by human landing catches (HLC) and light traps, respectively, and corresponding relative changes due to the interventions.

	Placebo control	Pull (Odour-baited Suna trap)	Push (Transfluthrin-treated fabric strip on roof eave gaps)	Push–pull (Combination of transfluthrin strip and odour-baited traps)
OUTDOOR Human landing collections				
*Anopheles funestus*	6.0 (2.5,10.9)	7.3 (2.9, 13.4),	8.9 (3.6, 16.1),	7.4 (3.0, 13.8),
		+ 21.9% (− 10.8%, + 56.5%)	+ *47.6% (*+ *11.2%,* + *85.3%)*	+ 22.6% (− 13.3%, + 62.2%)
*Anopheles arabiensis*	23.0 (12.9,35.1)	20.6 (11.7, 31.6),	24.3 (13.9, 37.1),	22.7 (12.9, 35.1),
		− 10.4% (− 23.2%, + 4.2%)	+ 5.7% (− 10%, + 21.3%)	− 1% (− 17.4%, + 17.4%)
*Culex*	46.4 (32.3,61.6)	40.2 (27.7, 54.3),	**19.3 (13.4, 26.2),**	**20.7 (13.5, 29.1),**
		− 13.1% (− 29.6%, + 3.7%)	**− 58.3% (− 65.9%, − 50.2%)**	**− 55.2% (− 66.5%, − 41.6%)**
*Mansonia*	10.3 (6.8,14.5)	9.7 (6.4, 13.6),	5.4 (3.5, 7.6),	5.6 (3.7, 8.1),
		− 5.1% (− 27.3%, + 16.7%)	**−47.1% (− 59.9%, − 34.5%)**	**− 45% (− 60.2%, − 30.6%)**
INDOOR light trap collections				
*Anopheles funestus*	33.4 (18.5,51.8)	31.0 (16.9, 49.1),	**11.8 (5.9, 19.3),**	**11.1 (5.8, 17.6),**
		− 6.6% (− 29.3%, + 17.8%)	**− 64.3% (− 75.7%, − 50.0%)**	**− 66.5% (− 75.7%, − 56.4%)**
*Anopheles arabiensis*	1.9 (1,3.1)	1.8 (0.9, 3),	1.1 (0.5, 1.9),	1.1 (0.5, 1.8),
		− 6% (− 39.3%, + 30.7%)	**− 41.3% (− 64.5%, − 17.5%)**	**− 44.3% (−65.5%, − 20.4%)**
*Culex*	2.2 (0.8,4.1)	2.2 (0.7, 4.3),	1.8 (0.4, 3.8),	0.8 (0.2, 1.7),
		+ 2% (− 41.5%, + 54.2%)	− 14.1% (− 65.6%, + 55.4%)	**− 60.2% (− 81.1%, − 37.7%)**

All results are expressed as the mean and 95% highest density credible interval (HDI) of the posterior distribution of the outcome, with interval estimates (HDIs) corresponding to an average house and an average week. A relative change has 95% credibility if its HDI excludes 0% (unity). Both, an intervention mean mosquito density whose 95% HDI is lower than and does not widely overlap with the corresponding control HDI, and a positive protective efficacy (negative relative change) with 95% credibility, is highlighted in bold. A negative protective efficacy (positive relative change) with 95% credibility is highlighted in italics. For example, outdoors, the mean Culex density under the push intervention (95% HDI (13.4,26.2)) was lower than the control mean Culex density (95% HDI (32.3,61.6)) with 95% credibility, and the push had a positive protective efficacy with 95% credibility (the 95% HDI (-65.9%, -50.2%) excludes 0%). As it is a relative measure, protective efficacy may well show an effect with 95% credibility (95% HDI excludes unity), even if the corresponding HDIs for the intervention and control mean mosquito densities do overlap. Thus, on the basis of 95% credibility, the mean Mansonia density under the push intervention (95% HDI (3.5,7.6)) was not completely separated from the control mean Mansonia density (95% HDI (6.8,14.5)), but the push still had a positive protective efficacy with 95% credibility for Mansonia (the 95% HDI (− 59.9%, − 34.5%) excludes 0%).

Contrary to the impact on malaria vectors, both the ‘push’ and the ‘push–pull’ had a strong and comparable protective efficacy reducing biting densities of *Culex* and *Mansonia* mosquitoes outdoors. The ‘pull’ alone was also without impact on these species (Table 1).

#### Indoors

The mean indoor density of primary malaria vectors per house-night in control houses was estimated as 33.4 (95% HDI: 18.5 to 51.8) for *An. funestus* and 1.9 (95% HDI: 1.0 to 3.1) for *An. arabiensis*. Under both, the ‘push’ and the ‘push–pull’ interventions, the indoor density of *An. funestus* strongly decreased with trivial difference between the two interventions, suggesting no added benefit from the ‘pull’ trap. The estimated protective efficacy was over 60% (Table 1). For *An. arabiensis* both interventions involving the ‘push’ showed also a protective efficacy indoors, however, the absolute density reductions were small. Similar to the results outdoors, the ‘pull’ showed no impact on mean indoor vector densities in the present trial. None of the interventions affected the dispersion of the indoor counts (see Supplementary Note). This is important as an increase of the dispersion due to an intervention would indicate a potential increase of heterogeneity in malaria transmission under the intervention.

Regarding *Culex* densities inside houses, the ‘push–pull’ treatment was associated with a decrease, while neither the ‘push’ nor ‘pull’ alone had a clear effect on indoor populations. *Mansonia* was excluded from this analysis as the indoor counts were too low to fit a model which was reasonably appropriate for the experimental design.

### Secondary outcome: Population removal of mosquitoes by the Suna trap

Efficient mass trapping of mosquitoes may diminish mosquito populations over time and thus may reduce biting rates beyond the reduction measured alongside trap deployment. However, as summarised in Table 2, the Suna trap (un-baited under the control and the ‘push’ intervention) caught very few mosquitoes for all species and across all experimental arms in the present study. Neither adding the bait to the Suna trap (‘pull’), nor the presence of the spatial repellent (‘push’), nor both together (‘push–pull’), influenced the Suna trap catches under a 95% credibility level.

**Table 2:** Mean Suna trap catches under the different interventions and corresponding relative changes with respect to the control.

	Placebo control	Pull (Odour-baited Suna trap)	Push (Transfluthrin-treated fabric strip on roof eave gaps)	Push–pull (Combination of transfluthrin strip and odour-baited traps)
**Suna trap collections**				
*Anopheles funestus*	1.4 (0.7,2.6)	2 (0.8, 4.6),	1 (0.4, 2.5),	1.3 (0.6, 2.9),
		+ 38.7%, (− 35.7%, + 168.7%)	− 27.1%, (− 65.6%, + 51.2%)	− 9.6%, (− 55.9%, + 73.4%)
*Anopheles arabiensis*	2.7 (1.5,4.5)	2.8 (1.5, 4.9),	2.4 (1.3, 4.1),	2.3 (1.2, 3.9),
		+ 4.7%, (− 30.7%, + 49.8%)	− 9.5%, (− 37.2%, + 22.2%)	− 16.1%, (− 44.2%, + 17.4%)
*Culex*	4.3 (2.2,8.1)	4.3 (2.1, 8.9),	4.7 (2, 10.8),	2.9 (1.3, 6.1),
		+ 0.3%, (− 38%0.6, + 55%)	+ 8.1%, (− 42.3%, + 97.4%)	− 33%, (− 61.3%, + 11.2%)
*Mansonia*	0.8 (0.2,2.6)	1 (0.2, 3.4),	0.9 (0.2, 4.4),	1.2 (0.2, 13.5),
		+ 13.5%, (− 56.2%, + 124%)	+ 4.8%, (− 72.3%, + 227.5%)	+ 31.9%, (− 78.6%, + 1022.2%)

All results are expressed as the median and 95% highest posterior density credible interval (HDI) of the posterior distribution of the outcome, with interval estimates (HDIs) corresponding to an average house and an average week. The median of the posterior distribution was chosen as the point estimate since the posterior distributions are very long-tailed to the right. No cells are highlighted here, since for none of the interventions and none of the species the Suna trap catches changed with 95% credibility as compared to the control (unbaited Suna trap).

### Variability in mosquito density and protective efficacy across houses and weeks: an arbitrary house-week analysis

The interval estimates presented above refer to an average situation, corresponding to a hypothetical house and week being the average over all the houses and weeks seen in the trial (average house-week). For a very large field study such average estimates would be very narrow (assuming the model to be well specified), which might mistakenly be interpreted as a uniform effect across houses and weeks. However, the hierarchical, multilevel Bayesian analysis also provides predictions of the mosquito density and the protective efficacy that is expected for an arbitrary house and week in the field (arbitrary house-week), based on the variability over houses and weeks seen in the field trial. The interval estimate for an arbitrary house-week will always show considerable uncertainty no matter how big the study is, reflecting the high variability of mosquito densities and of intervention effectiveness in reality. In other words, if potential users of the intervention ask how much it will reduce the mosquito density at their house, they should be communicated the interval estimates (HDI) for an arbitrary house-week (given no specific relevant information about them or their house is available), and not the interval estimate (HDI) for an average house-week (which may not even exist in the field). Interval estimates with respect to an arbitrary house-week are graphically displayed in Figs. 1 and 2 and reported in the Supplementary Table S1. Note that the difference between the average and the arbitrary house-week analysis lies in the uncertainty quantification given by the interval estimates (HDI), while the point estimates (means of the posterior distributions) are identical.

As shown by the left-hand side graphs in Figs. 1 and 2, the estimated distributions of the mean mosquito densities for an arbitrary house-week (red curves) carry much more uncertainty than the ones for an average house-week (blue curves), with the latter corresponding to the mean mosquito densities presented in Table 1. In the same charts, the actual mosquito density data (turquoise bar plots) and simulated mosquito density data sampled from the fitted model (coral bar plots) are plotted, confirming that the model represents the real data well (posterior predictive check). Estimates of the mean mosquito densities per specific house and week are contained in Supplementary Figs. S5 and S6, respectively.

As shown by the right-hand side charts in Figs. 1 and 2, the protective efficacy estimates for an arbitrary house-week (red curves) contain more variability than the ones for an average house-week (blue curves), but the discrepancy is smaller than for the mosquito densities. Importantly, the positive outdoor protective efficacies for culicine mosquitoes with 95% credibility in the average house-week case also have 95% credibility under the arbitrary house-week view, while the finding that the push increased *An. funestus* densities outdoors has less than 95% credibility regarding an arbitrary house-week. Indoors, the positive protective efficacies of ‘push’ and ‘push–pull’ against *An. funestus* retained 95% credibility under the arbitrary house-week view. However, for *An. arabiensis* and culicine mosquitoes, with low baseline indoor densities, none of the interventions showed a positive predictive efficacy of at least 95% credibility indoors with regard to an arbitrary house-week. In summary, the protective efficacies of ‘push’ and ‘push–pull’ were fairly uniform across houses and weeks. This finding is also supported by the fact that a model assuming no variation of the intervention effect across houses or weeks had similar predictive accuracy to the chosen model (see Supplementary Methods).

## Discussion

Our field study revealed a very high malaria vector density in rural communities within the well-established Ahero rice irrigation scheme in western Kenya. Notably the sampling tools employed, highlighted a nearly balanced vector host-seeking density outdoors and indoors, with a mean of 29–35 estimated bites per person per night in either location, during the short rainy season. The outdoor malaria vector population was dominated by *An. arabiensis* and the indoor population by *An. funestus*. Such observations have been reported in other studies^31–33^; yet traditionally, vector densities have been largely monitored indoors. Our findings highlight the need for outdoor surveillance, so that the actual malaria transmission risk is not underestimated. It further emphasises the need for efficient outdoor sampling tools with comparable efficiency of the human landing catches. Light traps used in the outdoors tend to highly underestimate actual vector densities^34,35^.

Our findings also corroborate the need for vector control interventions targeting the outdoor peri-domestic space. However, none of the three tested interventions, transfluthrin-treated eave strips (‘push’), odour-baited trap (‘pull’) and the combined ‘push–pull’ system, reduced outdoor biting by either of the two abundant malaria vectors, *An. arabiensis* and *An. funestus*. On the contrary, the presence of a spatial repellent was associated with an increase of *An. funestus* biting outdoors.

There was, however, a strong protective efficacy of the ‘push’ and ‘push–pull’ intervention for the indoor environment. Indoor vector densities were significantly reduced to a similar extent by both interventions. There was no benefit to combining the ‘push’ with the ‘pull’ as seen by either an increased protective efficacy or an increase in the mosquito population removed by the ‘pull’. *Anopheles funestus* densities indoors were reduced by around 60% in the presence of transfluthrin. The relative reduction of indoor densities of *An. arabiensis* was significant in the analysis for an average house-week, but not in the analyses for an arbitrary house-week. This might warrant further studies given that the absolute risk reduction from this species in our study location was small due to generally low indoor densities.

These findings from the field stand in contrast to preceding semi-field evaluations in large field cages from western Kenya^8,9^ which suggested outdoor protection against bites from *An. arabiensis* for the transfluthrin-based intervention. This observation emphasizes the importance of field evaluations of novel interventions that have been developed under more standardized conditions, prior to advocating for larger scale use. The preceding semi-field results, however, were also highly variable^8^ with low efficacy (less than 20% protection) observed at low evening temperatures^10^. Our field trial was implemented during the rainy season, when malaria transmission tends to peak in western Kenya, but average night-time temperatures are low, ranging between 16 and 20 °C^36^. For passive emanation from treated fabric, it has been demonstrated that the airborne concentrations of transfluthrin decreases with decrease of temperature^37^. Climate variation might potentially also explain the contrasting results from south-eastern Tanzania where semi-field evaluations with the same spatial repellent suggested good outdoor protection^14,15,23^ and where impact was shown in experimental hut trials on indoor and outdoor vector densities^23^. However, not only was the average night-time temperature during these field experiments higher, but also the transfluthrin load applied was 7-times higher^23^ than what we used in our field trial. Our test load was informed by proceeding semi-field evaluations, which suggested good efficacy at release rates that would be save for consistent human exposure^8,18^.

The increase of *An. funestus* bites outdoors under the ‘push’ and ‘push–pull’ interventions might have been due to diverting of indoor biting to outdoor biting, especially as high numbers of *An. funestus* were prevented from house entry. However, further studies are required to establish such causality, especially since this effect had less than 95% credibility in the arbitrary house-week analysis. The protective efficacy of the transfluthrin-treated fabric strip around the eave gaps on indoor densities in the field was in the same range as shown under semi-field conditions in western Kenya^10^ and Tanzania^14,15,23,38^. Whilst semi-field experiments were done without strong air movement in the systems^8^, the field trial presented here, was implemented at the eastern shores of Lake Victoria, a region characterised for its strong evening breeze driven by the thermal gradient between the lake and land surface^39^. These conditions likely diluted and dispersed any airborne concentration of transfluthrin in the outdoor space quickly. Such climatic impact is less expected indoors and the passively released transfluthrin might hence have accumulated. Furthermore, for entering the house through the open eave gaps under the roof^40,41^, mosquitoes need to pass closely by the treated fabric strips where the concentration may be highest, all of which might explain the sharp contrast between indoor and outdoor protection. The impact of climate conditions, such as night temperatures and winds, on the transfluthrin-based intervention requires further investigations, with experiments specifically designed to answer this question. The climate conditions during the here presented trial were too consistent to analyse any seasonal effects. Such data could then be used to better model the eco-epidemiological settings where such interventions would have the biggest impact.

The odour-baited Suna trap neither reduced mosquito bites and house entry, nor did it catch mosquitoes in any biologically relevant numbers to support a vector control effort by reducing vector densities through mass-trapping. Given the high mosquito densities overall, the powered traps only coincidentally caught *Anopheles* mosquitoes (on average 1–2 individuals per night), regardless of the presence of bait (MB5 and 2-butanone) or the presence of the repellent. No synergistic effect between bait and repellent (by diverting mosquitoes to the Suna trap) in terms of Suna trap catches was detected. This finding is consistent with the preceding semi-field study^8^ with *An. arabiensis* as test organisms but stands in contrast to the success of a mass trapping intervention with exactly the same trap and bait in a cluster randomised controlled trial on Rusinga island, western Kenya^27^, where *An. funestus* indoor density reductions by two thirds were associated with the intervention. This trapping success might, in part, have been a result of the location of the trap placement, which was in contrast to our study, directly under the open eave gaps of houses, presumably benefitting from the odour plume coming through the roof gaps of the house^42–44^. Mosquitoes may therefore have been attracted to the house by the host cues including carbon dioxide and only subsequently be trapped due to the artificial lures. Recent studies have re-emphasized the important role of carbon dioxide supplementation to odour mixture on catches of *An. arabiensis* and *An. funestus* with odour-baited traps^45,46^. For logistical reasons, however, carbon dioxide was not used to complement the chemical blend in this study and is unlikely to be feasibly used in large-scale interventions. Notably, the mass-trapping trial on Rusinga island was not able to establish an impact on *An. arabiensis* densities, suggesting species specific responses that might need further evaluations^8,27^.

In our study site, the mean nightly indoor collections of *An. funestus* were over five times higher than outdoors. This could be due to an innate preference for feeding indoors even when a human host was exposed outdoors, or preferential biting for later in the night when no humans were exposed outside, since our human landing collections stopped at 23:00 h. A limitation of our study, which should be taken into consideration when interpreting the results, is the fact that we have used different mosquito sampling methods outdoors and indoors. Human landing catches, which were used for outdoor sampling, represent the best proxy measure for mosquito biting, and likely is more efficient than light trap collections which were used indoors^35^. However, HLCs are prone to human errors, require tight supervision for adherence to protocols, are very intrusive and disruptive to people’s privacy in the home, and are very tiring, hence in our study we only implemented them outdoors for five hours from 18:00 to 23:00 h. We consequently do not have any outdoor biting measures for later night hours or early mornings and might have underestimated the outdoor vector population. The light trap collections, however, ran for 12 h throughout the night. Whilst the indoor trap collection gives an idea as to how many mosquitoes entered the indoor space to seek hosts, it cannot exactly estimate how many might have bitten, especially when people are protected by a mosquito net. Using our approach, we are not able to establish to what extent indoor exposure to transfluthrin might have affected survival of indoor mosquitoes as it has been demonstrated that at higher airborne concentrations transfluthrin can lead to knockdown and mortality^47^.

Whilst not the focus of our study, it is important to highlight that outdoor densities of *Culex* and *Mansonia* mosquitoes were reduced by 60% and 50%, respectively, by the spatial repellent. This is especially noteworthy, given the high biting burden from these vectors outdoors in the study location. Transfluthrin-treated eave strips might therefore be a promising tool to protect from nuisance biting and for the control of lymphatic filariasis as well as arboviral zoonotic diseases^48,49^, in an integrated vector management approach^12^. This important effect may also encourage uptake and compliance with the intervention. *Culex* and *Mansonia* bite earlier in the evening when temperatures are still elevated, as opposed to *Anopheles* mosquitoes, which might, in part, explain why these species were repelled in the outdoor space, however, further studies are required to establish the precise mode of action of this intervention on different mosquito species in different ecologies.

Our hierarchical Bayesian modelling framework provides easily interpretable interval estimates for both the control and the intervention effect across different houses and weeks. Despite the selection of relatively uniform households along a transect with all houses facing the flooded rice fields, mosquito densities were highly variable across houses and weeks. This might have been associated with a range of biotic and abiotic factors out of our control, such as distance to productive larval microhabitats^50^, differential attractiveness of house residents^51,52^, livestock density in and around the location^53^, and weather conditions (rain, wind, humidity). Nevertheless, as estimated by the model, the intervention effects were relatively uniform. However, since we purposely selected very similar houses close to mosquito breeding sites, it is likely that variability between sampled houses was less than that in the whole population, but this does not imply a bias in the intervention effect. The model estimated no increase of the dispersion of mosquito counts due to the interventions and thus the protective efficacy measure relying on mean densities seems to be a good measure to adequately characterise the intervention effect. Hence, our model generated robust estimates of the protective efficacy and highlighted the influence of the natural variability, enabling a more cautious evaluation of the intervention. A limitation of our modelling approach is the fact that we did model the push–pull intervention as a single intervention package rather than as push, pull and an interaction between the two components. However, even though estimating the interaction would be interesting and instructive for the further development of push–pull systems, this would not alter our conclusions in terms of the interventions’ public health relevance in the indoor and outdoor environment. By use of a simulation-based model of malaria epidemiology informed by this Bayesian inference, the impact of a large-scale implementation of the considered interventions on malaria case incidence can be estimated whilst considering the full uncertainty of the inference.

## Conclusion

In a rice irrigation setting with a high burden of *An. funestus* seeking hosts indoors and a similarly high burden of *An. arabiensis* seeking hosts outdoors, we could demonstrate a strong protective efficacy of transfluthrin-treated fabric strips positioned along eave gaps of rural houses against indoor biting but failed to achieve any protection from outdoor biting malaria vectors. Additional indoor protection is desirable especially during the early evening and morning hours when people are not protected by bednets^54^ and based on our results, a spatial repellent could be a valuable complementary tool. The intervention might also warrant further study in settings where coverage and/or use of insecticide-treated nets is insufficient for varied social and cultural reasons. The passively released spatial repellent did not protect against outdoor biting by malaria vectors, presumably because of a low airborne concentration of transfluthrin in the outdoor space due to a low evaporation rate and quick dilution. The need for finding highly efficient methods for protecting people from malaria vector bites outdoors remains important, as illustrated in our study, where a human landing volunteer received on average 23 bites from *An. arabiensis* per night during the 17 weeks of survey during the rainy season. With 1% of the collected *An. arabiensis* being *Plasmodium* sporozoite positive, this would result in more than one infective bite per person per week. Whilst the more endophilic *An. funestus* might in principle be the more competent vector^55^, its role in malaria transmission in the study area will be confounded by the promotion and use of bednets indoors, whilst there are no widely available outdoor interventions available. The odour-baited Suna trap provided neither an indoor nor an outdoor protection and showed no relevant killing effect, regardless of whether being deployed alone or in combination with the spatial repellent. The reason for adding an attractive trap to the spatial repellent was to avoid diverting bites to others, and in fact, our results suggest that repelling vectors from the indoor space might have resulted in an increased biting in the outdoor space by *An. funestus*. Hence there remains an urgent need to further develop and evaluate odour-baited attract-and-kill approaches that can be effectively combined with spatial repellents for a push–pull intervention for malaria control.

## Methods

### Experimental design

Four treatment arms (‘push–pull’, ‘pull’ alone, ‘push’ alone and placebo control) were allocated in a randomised block design to 12 houses. The treatments were randomly rotated through the houses so that each house received all four treatments. Mosquito sampling was done over four consecutive nights a week and treatments were rotated on a weekly basis. For each house to receive all four treatments a complete round required four weeks. Four complete rounds and one additional week (total 17 weeks) were implemented.

For the ‘push’ intervention, transfluthrin (Bayer Global, Leverkusen, Germany) emulsified concentrate (EC) of 0.2 g/ml was applied on hessian fabric to achieve a load of 2.5 g/m^2^^8,10^. The treated fabrics were cut into strips measuring a length of 24 m, corresponding to the perimeter of the eave gaps of the houses, and a width of 0.05 m. Correspondingly, untreated fabric strips were prepared in the same dimensions and placed around the eave gaps on the control houses as well as on the houses with the push only intervention to account for any potential mechanical obstruction by the fabric for house-entering mosquitoes, hence allowing us to exclusively assess the impact of the active ingredient. Based on data from semi-field experiments assessing the suitability of fabric strips, however, we do not expect that the untreated fabric strip had any impact on indoor densities^10^. The fabric strips were fixed with flexible aluminium wires covering the eave gap partially, leaving a similar space above and below to allow for movement of air^10^. New fabric strips were used for every weekly rotation.

Odour-baited Suna traps were used as pull devices. The trap’s development, appearance and operation are described in detail elsewhere^29^. The odour bait was a synthetic chemical blend (MB5) comprised of ammonia (2.5% in water), l-(+)-lactic acid (85% in water), tetradecanoic acid (0.00025 g/l in ethanol), 3-methyl-1-butanol (0.000001% in water), and butan-1-amine, prepared at a concentration of 0.001% in paraffin oil and presented on nylon strips^56^. An additional nylon strip was treated with 2-butanone which has been proposed as a replacement for CO_2_ previously^57^. The trap was suspended using a tripod 5 m away from the house’s main entrance with the main odour-release point approximately 0.3 m off the ground. Traps were placed in all treatment arms and run nightly with the fan switched on, however, the nylon strips were untreated in the control and ‘push’ only houses, whilst treated strips were inserted in the traps in the ‘pull’ only and in the ‘push–pull’ intervention arms.

All field workers, the community members, and the investigators implementing the mosquito identification, were blinded to the treatment allocation. The project manager (MMN) prepared the hessian fabric and nylon strips and labelled them with a unique identifier according to the treatments. She allocated the treatment to the houses using a random number generator and sent the weekly allocation information and the fabrics and strips to the field, where the site leader oversaw the installation. Houses, traps, and treatments were coded, and the code was only revealed after initial analysis**.** The initial data exploration and analyses was also done blinded but consequently the labels were opened for the final Bayesian statistical modelling.

### Study site

**Figure 3:**
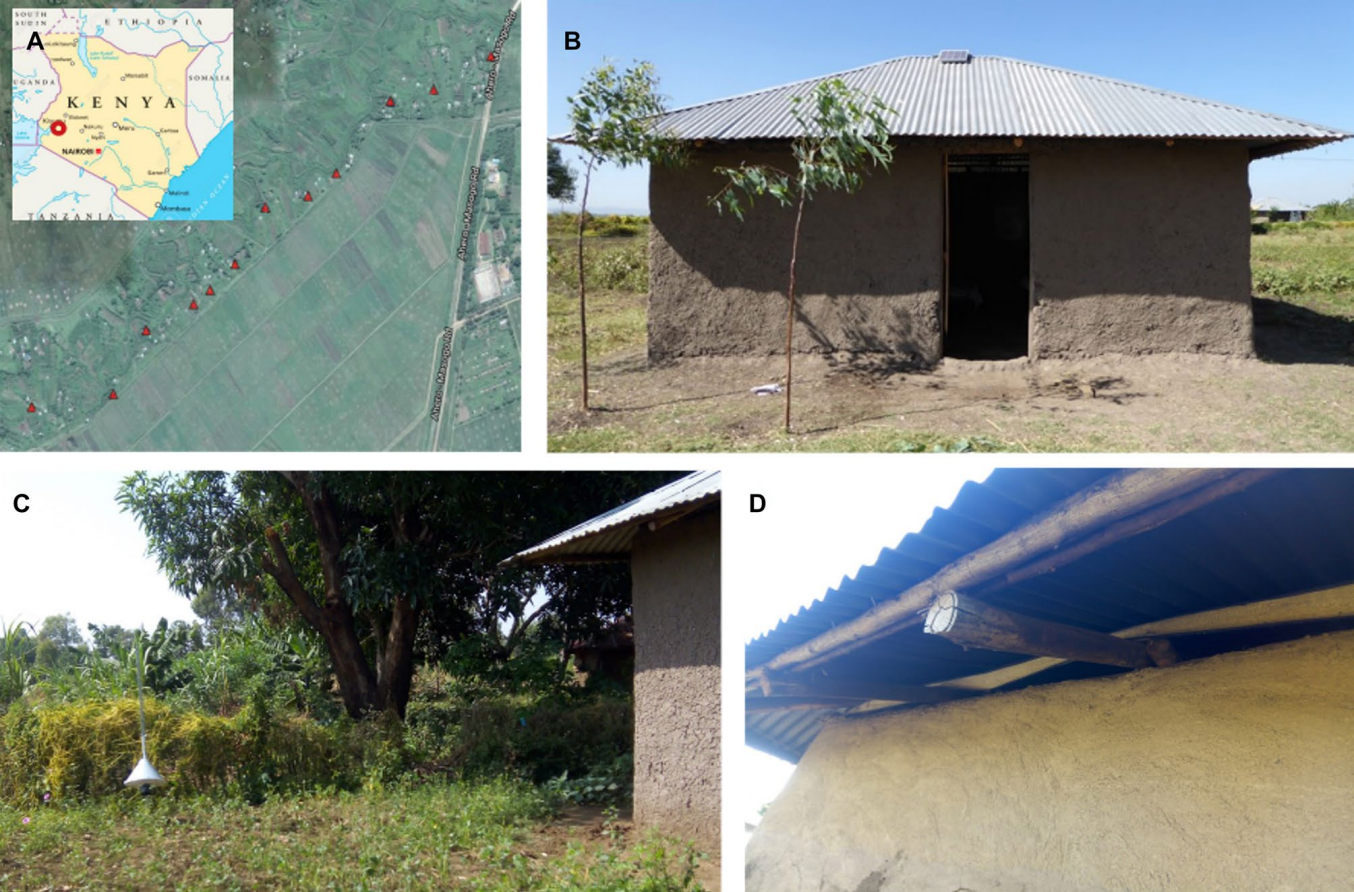
Study location and treatment set-ups in Ahero rice irrigation scheme, western Kenya. (**A**) Red triangles show the transect of selected trial houses; (**B**) Traditional mud huts of families enrolled for the study; (**C**) Suna mosquito trap located 5 m away from house; (**D**) Hessian fabric strip allocated to eave gap under the roof.

The study took place within the Ahero rice irrigation scheme (Kisumu County, Muhoroni sub-county; 0°05′30 S 34°59′40 E) between September and December 2018. Twelve houses of similar traditional build (single room, 36 m^2^, mud walls, iron sheet roof, open eave gaps) were purposely selected along a transect of 2.5 km. The houses were at least 200 m apart and were all facing the irrigated rice fields (Fig. 3). All houses were occupied by two adults. New Olyset® (Sumitomo Chemical Ltd) bed nets were provided to all households before the start of the trial.

### Human landing collections outdoors

For collecting mosquitoes outdoors using the human landing collection method (HLC)^30^, male volunteers between 28 and 61 years of age were recruited and trained. These volunteers were either residents of the selected 12 houses or selected by the household heads of the enrolled houses to ensure that the human landing volunteer had the trust of the occupants of the homestead. Volunteers were screened for malaria parasites weekly using rapid diagnostic tests, no infections were registered during the trial. Collections were implemented from 1800 to 2300 h from Monday to Thursday every week over 17 weeks. Human landing volunteers were fixed to study houses and so variation between volunteers (individual attractiveness, collection skills) is tied to the house. Indoor presence during the outdoor HLC collections was not systematically recorded. However, since the residents lived their normal lives, it can be assumed that people were present indoors during the outdoor HLC collections.

### Light trap collections indoors

CDC light traps (John W Hock, USA) were set inside the house positioned at the foot end of the bed with the cover section approximately 15 cm above the height of the mattress^30^. Residents were protected by a bed net while sleeping. Trapping was done from 1800 to 0800 h on the same nights as the HLC took place outdoors.

### Mosquito identification and sporozoite infection

All collected females were separated morphologically to genus level and all *Anopheles* specimens further identified to species level^58^. Molecular analyses were done to identify the proportional contribution of sibling species in the species complexes and to investigate the sporozoite infection rates in the major vectors indoors and outdoors to estimate the potential impact of the interventions on malaria transmission. A random sub-sample per sampling location and month of 36% (n = 11,920) of all collected specimens of the *An. gambiae* and *An. funestus* complexes from outdoor HLC and indoor trap collections were analysed. Sibling species in species complexes were separated using PCR-based methods^59^. Sporozoite analysis was done on pools of 10 females using SYBR Green real-time PCR restriction fragment length polymorphism assay (cytb-qPCR) targeting the cytochrome b gene of the four major human *Plasmodium* species (*P*. *falciparum*, *P*. *malariae*, *P. vivax* and *P*. *ovale*)^60^.

### Sample size considerations

Initial sample size calculations were performed with respect to a generalized linear mixed model and significance level of 0.05 for rejecting the null hypothesis, by use of a simulation-based power analysis^61^ with one thousand simulations in R statistical software^62^. Based on previous mosquito collections at the same field site, we assumed a mean catch size of 30 malaria vectors per night in the placebo control arm Variation at the house and week level was set at 50%, each, and Latin square replication variation was set at 50%. Given these parameters, the Latin square study design described above had > 80% power to detect an impact of the intervention of at least 23%.

### Data processing

Each data point consists of an outdoor (HLC) and indoor (CDC light trap) mosquito count as well as a Suna trap catch count. HLC only attracted host-seeking, female mosquitoes, while the CDC light trap caught a small number of males and fed females (< 5% of catch). Males and fed females were excluded from the analysis for comparability given that the interventions were aimed at reducing potentially infectious bites from host-seeking females.

### Hierarchical Bayesian statistical model

Mosquito counts were analysed with a negative binomial model, with separate inference for outdoor, indoor, and Suna trap catch data. Model equations are provided in the Supplementary Methods. Each intervention was modelled as an additive effect on the log-scale on the mean of the negative binomial distribution, and as an alternation of the dispersion of the negative binomial distribution.

As data points collected at the same house or during the same week are not independent, a hierarchical second-level model^63^ was used to account for the dependency (grouping) between the data points in order to accurately quantify the uncertainty of the estimates. In particular, group-specific error terms (random effects) were introduced to allow the mean mosquito count to vary by both the house and the week, and, additionally,, the intervention parameter was allowed to vary by either house or by week. Both baseline and intervention parameters remained identifiable since, by design, for each house and each week both intervention and control data points were available. However, to prevent the intervention parameter from becoming unidentifiable it was not allowed to vary by both house and week simultaneously. For the dispersion parameter, no hierarchical second-level model structure was deployed (see the Supplementary Methods for an alternative model). Consequently, the fitted model includes three levels of indeterminacy of mosquito bite counts: variability of counts as described by the negative binomial distribution; variability of the parameters of the negative binomial model across houses and weeks, as described by the second-level model; and uncertainty of the parameter estimation, as described by the posteriors.

### Inference

For all parameters, uninformative prior distributions were chosen. All models were fitted by computational Bayesian inference with Rstan^64^, a probabilistic programming language implementing Hamiltonian Monte Carlo (HMC)^65^. All models were run for 2,000 iterations on each of six chains, with 1,000 of each chain’s iterations discarded as “warm-up”. All model parameters had $\widehat{R}<1.1$, providing evidence of convergence. Two models allowing for variation of the intervention parameter by house and by week, respectively, were averaged with equal weights to account for all possible dependencies introduced by the experimental design. This corresponds to more conservative interval estimates as compared to using a single model.

### Model assessment and diagnostics

In addition to the two models used for the final inference, different model variants were fitted in order to investigate the uniformity of the intervention effect (different hierarchical structures), the relation between indoor and outdoor biting (coupling indoor and outdoor) and dynamically increasing baseline mosquito density due to increasing rainfall over the course of the study. All models were compared by leave-one-out cross-validation (LOO-CV)^66^ by use of the R package ‘loo’^66^.

Estimates per specific house and week are provided in Supplementary Figs. S5 and S6, respectively. Detailed visual posterior predictive checks per house and week were conducted, and are provided as Supplementary Figs. S1–S4. It has been checked that no critical correlation between parameters occurred by visually checking pairwise scatter plots of the posteriors for all critical parameter pairs.

### Ethics approval and consent to participate

Approval for this study was obtained from the Scientific and Ethical Review Unit (SERU) of the Kenya Medical Research Institute (KEMRI): Non-KEMRI Protocol no. 621. All research was performed in accordance with the relevant guidelines and regulations. Informed consent was sought from all of the household heads and human landing catch volunteers.

## Supplementary Information

Supplementary Figure S1.

Supplementary Figure S2.

Supplementary Figure S3.

Supplementary Figure S4.

Supplementary Figure S5.

Supplementary Figure S6.

Supplementary Information 1.

Supplementary Information 2.

Supplementary Tables.

## Data Availability

The datasets analysed during the current study and all modelling codes are available from the corresponding author on reasonable request.
