# Supplementary material for: A randomized, double-blind placebo-control study assessing the protective efficacy of an odour-based ‘push–pull’ malaria vector control strategy in reducing human-vector contact
Source: Sci Rep. 2023 Jul 11;13:11197. doi: 10.1038/s41598-023-38463-5 (PMC10336143; doi:10.1038/s41598-023-38463-5)
Supplement: Supplementary file 3 — Supplementary Figure S3. [file 41598_2023_38463_MOESM3_ESM.pdf]

Supplementary Figure S3

to **"A randomized, double-blind placebo-control study assessing the protective efficacy of an odour-based 'push-pull' malaria vector control strategy in reducing human-vector contact"** by Ulrike Fillinger, Adrian Denz, Margaret M. Njoroge, Mohamed M. Tambwe, Willem Takken, Joop J.A. van Loon, Sarah J. Moore, Adam Saddler, Nakul Chitnis, Alexandra Hiscox

Visual posterior predictive check per house for indoor densities of *An. funestus* (A), *An. arabiensis* (B) and *Culex* (C).

Real data (blue) and simulated data (red), stratified by treatment (columns) and house (rows). For each house, the simulated data is with respect to an arbitrary week and hence shows the full variability over weeks. Note that, due to the averaging of two models with intervention location parameter varying either by house or week, for half of the simulated data points the intervention location parameter depended on the house and for half of the simulated points it didn't. The last row ('HOUSE 13') shows the simulated bite counts for an arbitrary house and arbitrary week and the real data pooled across all houses and weeks of the trial. Note that the last row ('HOUSE 13') is identical to the posterior predictive checks contained in Figures 1 and 2 of the main text.

A *An. funestus*

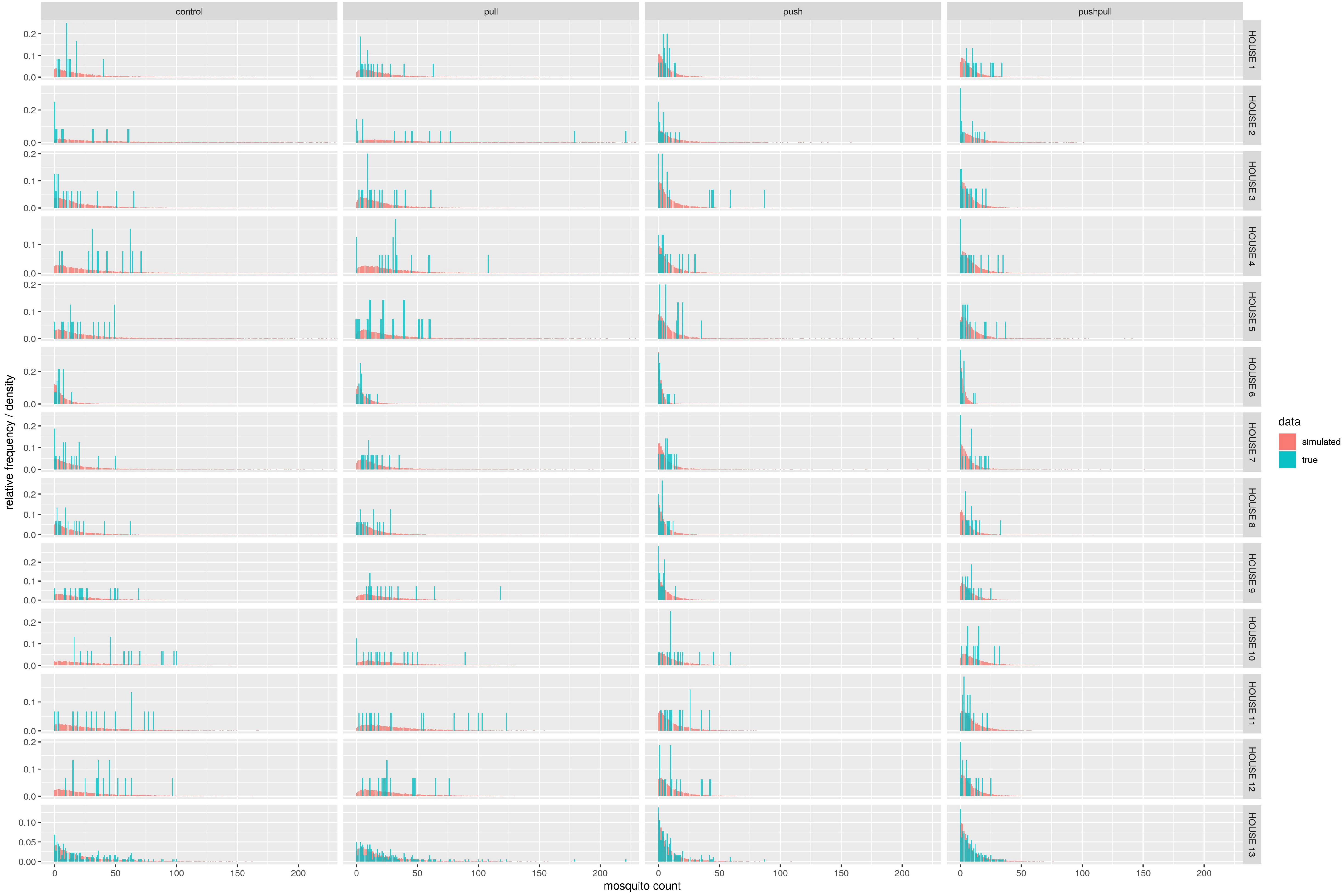

B An. arabiensis

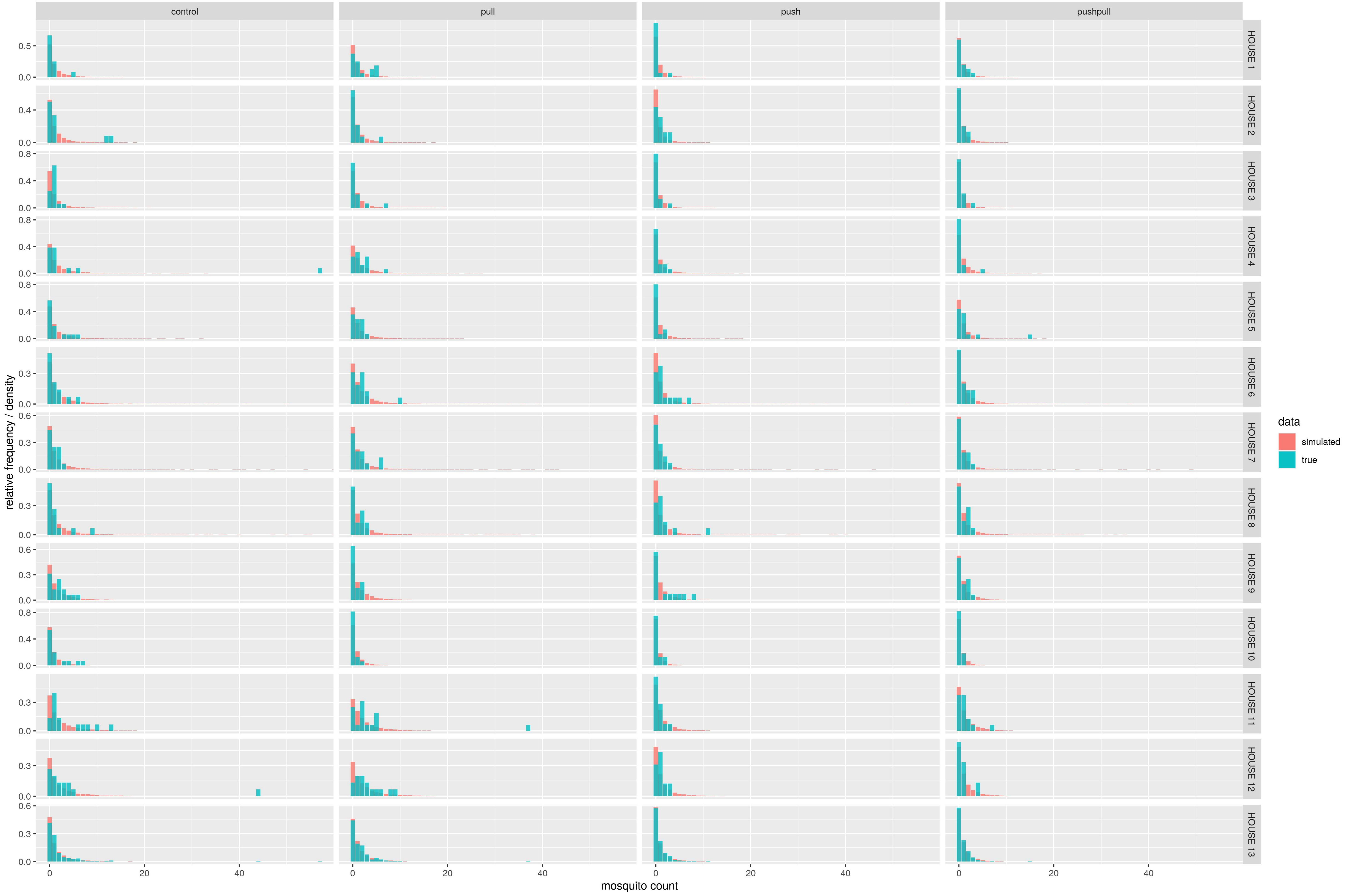

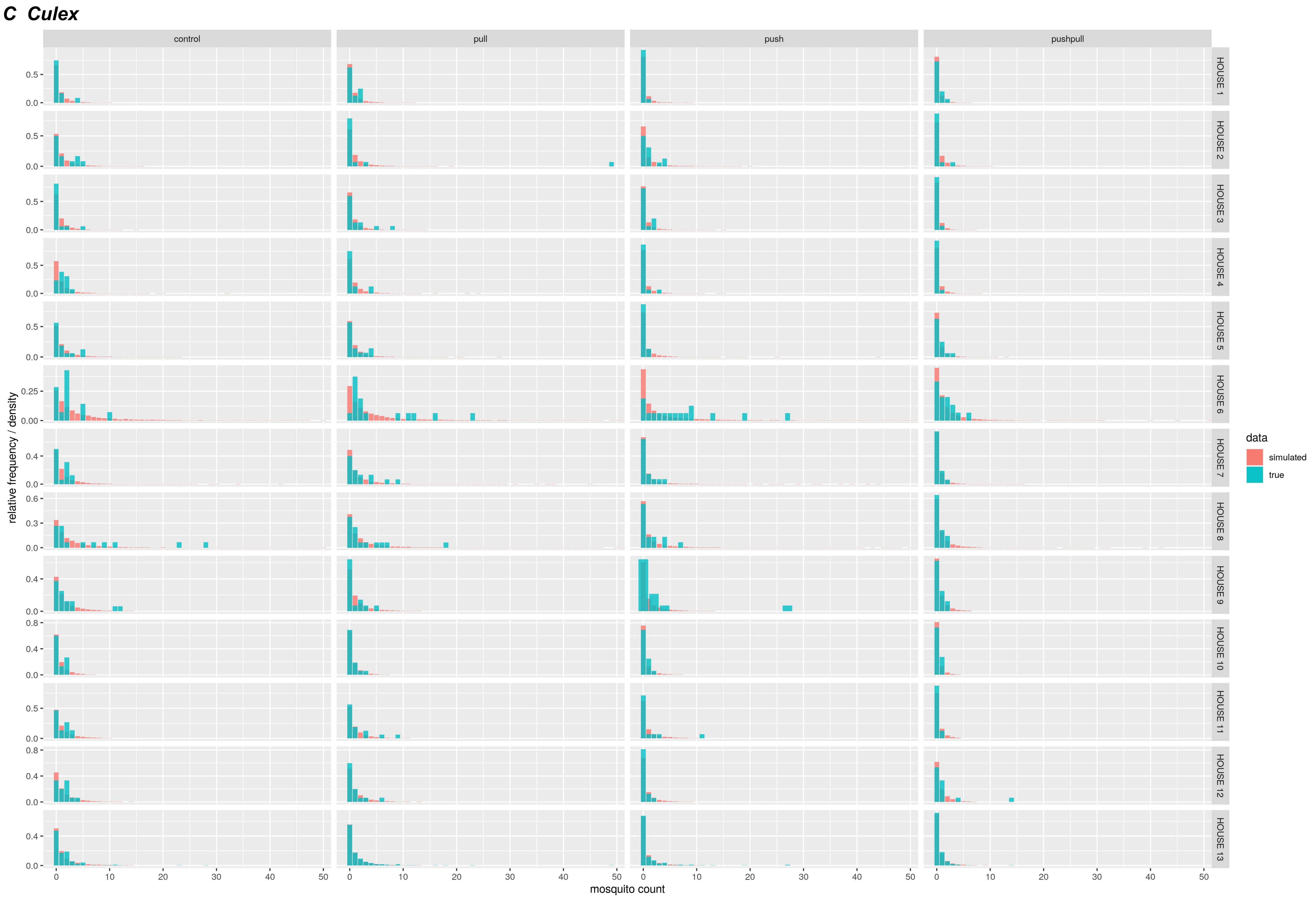

C Culex
control
pull
push
pushpull
HOUSE 1
HOUSE 2
HOUSE 3
HOUSE 4
HOUSE 5
HOUSE 6
HOUSE 7
HOUSE 8
HOUSE 9
HOUSE 10
HOUSE 11
HOUSE 12
HOUSE 13
relative frequency / density
mosquito count
data
simulated
true
