# Supplementary material for: A randomized, double-blind placebo-control study assessing the protective efficacy of an odour-based ‘push–pull’ malaria vector control strategy in reducing human-vector contact
Source: Sci Rep. 2023 Jul 11;13:11197. doi: 10.1038/s41598-023-38463-5 (PMC10336143; doi:10.1038/s41598-023-38463-5)
Supplement: Supplementary file 4 — Supplementary Figure S4. [file 41598_2023_38463_MOESM4_ESM.pdf]

Supplementary Figure S4

to **"A randomized, double-blind placebo-control study assessing the protective efficacy of an odour-based 'push-pull' malaria vector control strategy in reducing human-vector contact"** by Ulrike Fillinger, Adrian Denz, Margaret M. Njoroge, Mohamed M. Tambwe, Willem Takken, Joop J.A. van Loon, Sarah J. Moore, Adam Saddler, Nakul Chitnis, Alexandra Hiscox

Visual posterior predictive check per week for indoor densities of *An. funestus* (A), *An. arabiensis* (B) and *Culex* (C).

Real data (blue) and simulated data (red), stratified by treatment (columns) and week (rows). For each week, the simulated data is with respect to an arbitrary house and hence shows the full variability over houses. Note that, due to the averaging of two models with intervention location parameter varying either by house or week, for half of the simulated data points the intervention location parameter depended on the week and for half of the simulated points it didn't. The last row ('WEEK 18') shows the simulated bite counts for an arbitrary week and arbitrary house and the real data pooled across all houses and weeks of the trial. Note that the last row ('WEEK 18') is identical to the posterior predictive checks contained in Figures 1 and 2 of the main text. In WEEK 5, no data was collected, hence the corresponding row contains only simulated data (identical to the last row for an arbitrary week).

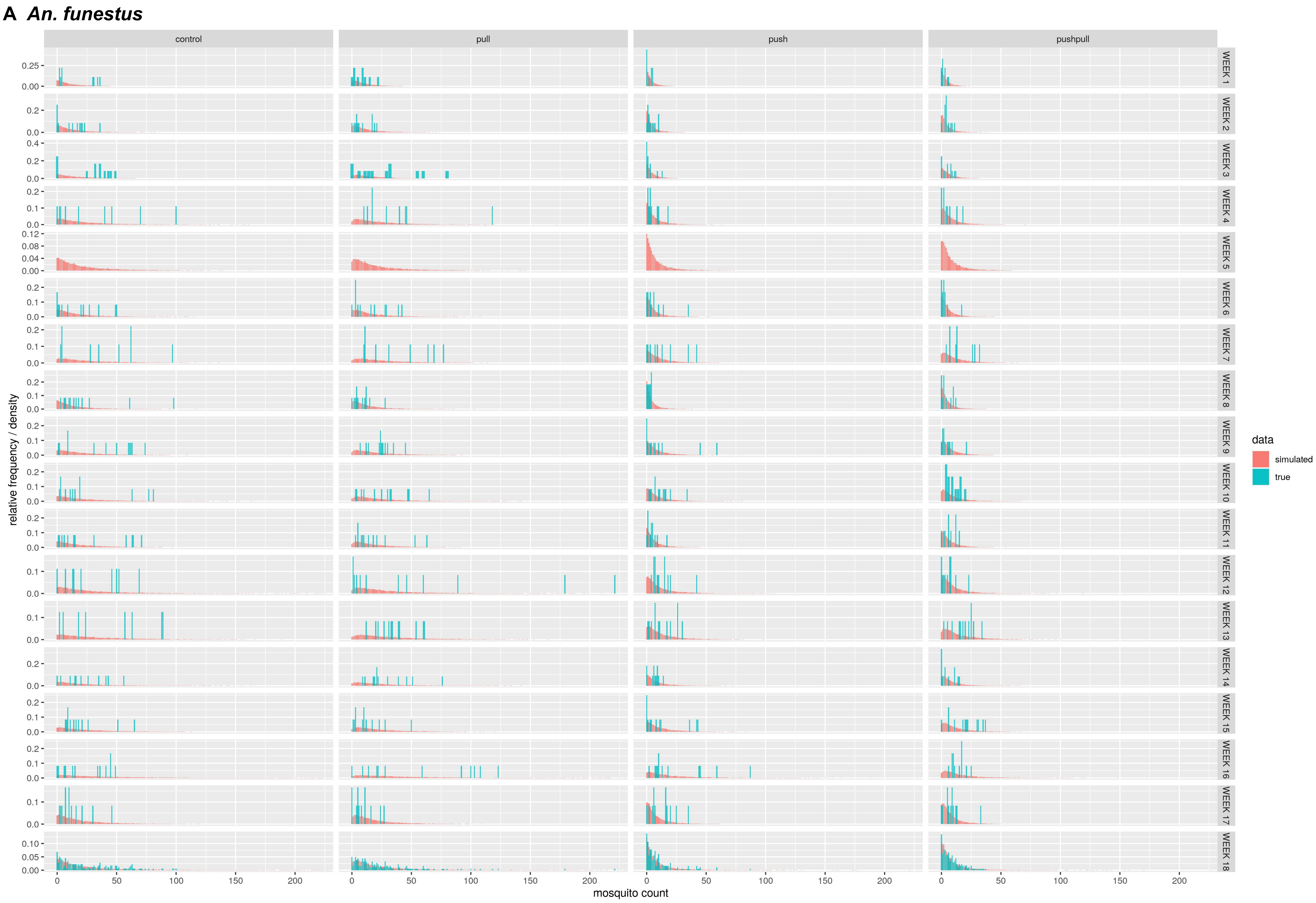

A An. funestus
control
pull
push
pushpull
WEEK 1
WEEK 2
WEEK 3
WEEK 4
WEEK 5
WEEK 6
WEEK 7
WEEK 8
WEEK 9
WEEK 10
WEEK 11
WEEK 12
WEEK 13
WEEK 14
WEEK 15
WEEK 16
WEEK 17
WEEK 18
relative frequency / density
mosquito count
data
simulated
true

B An. arabiensis

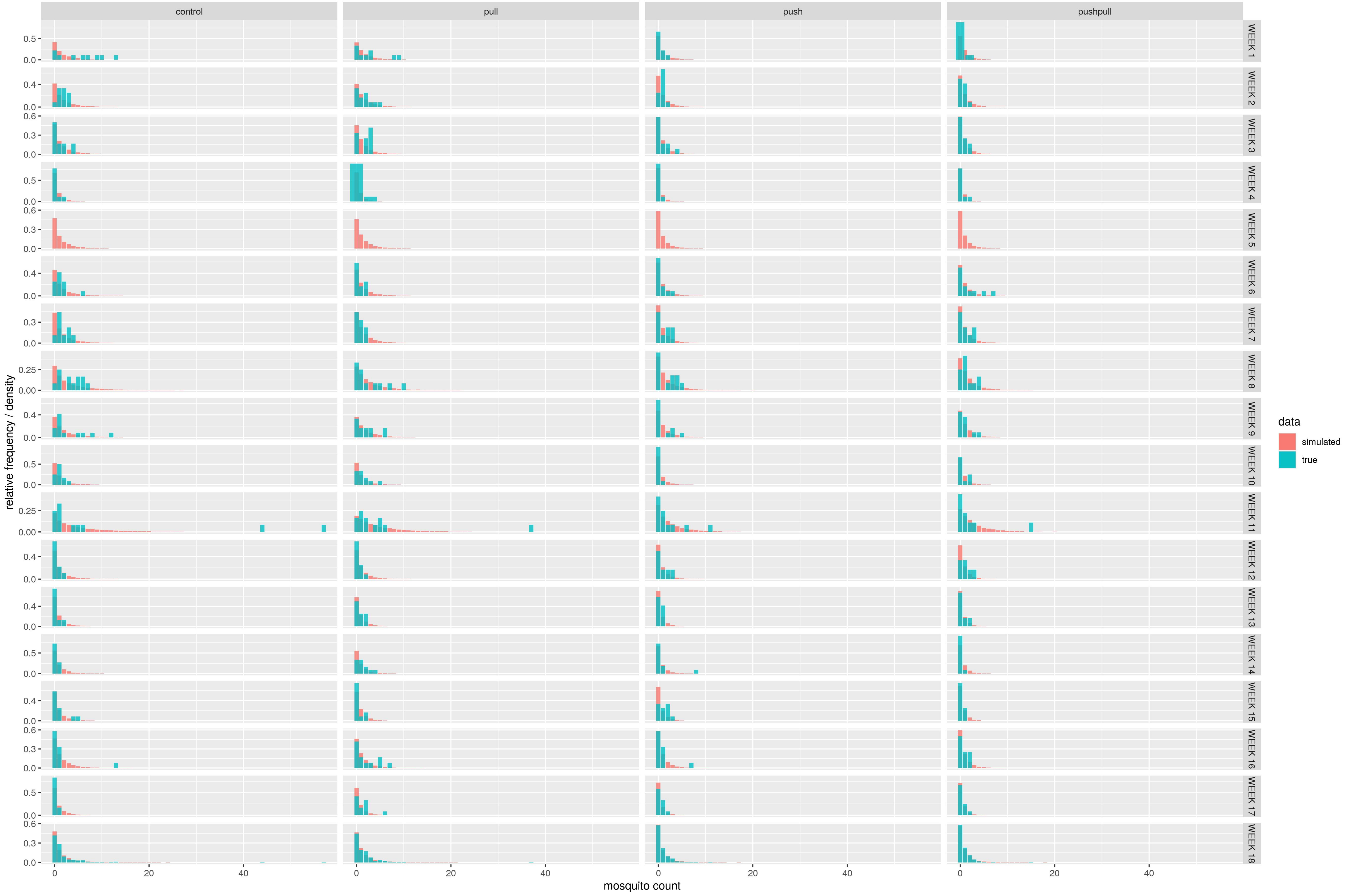

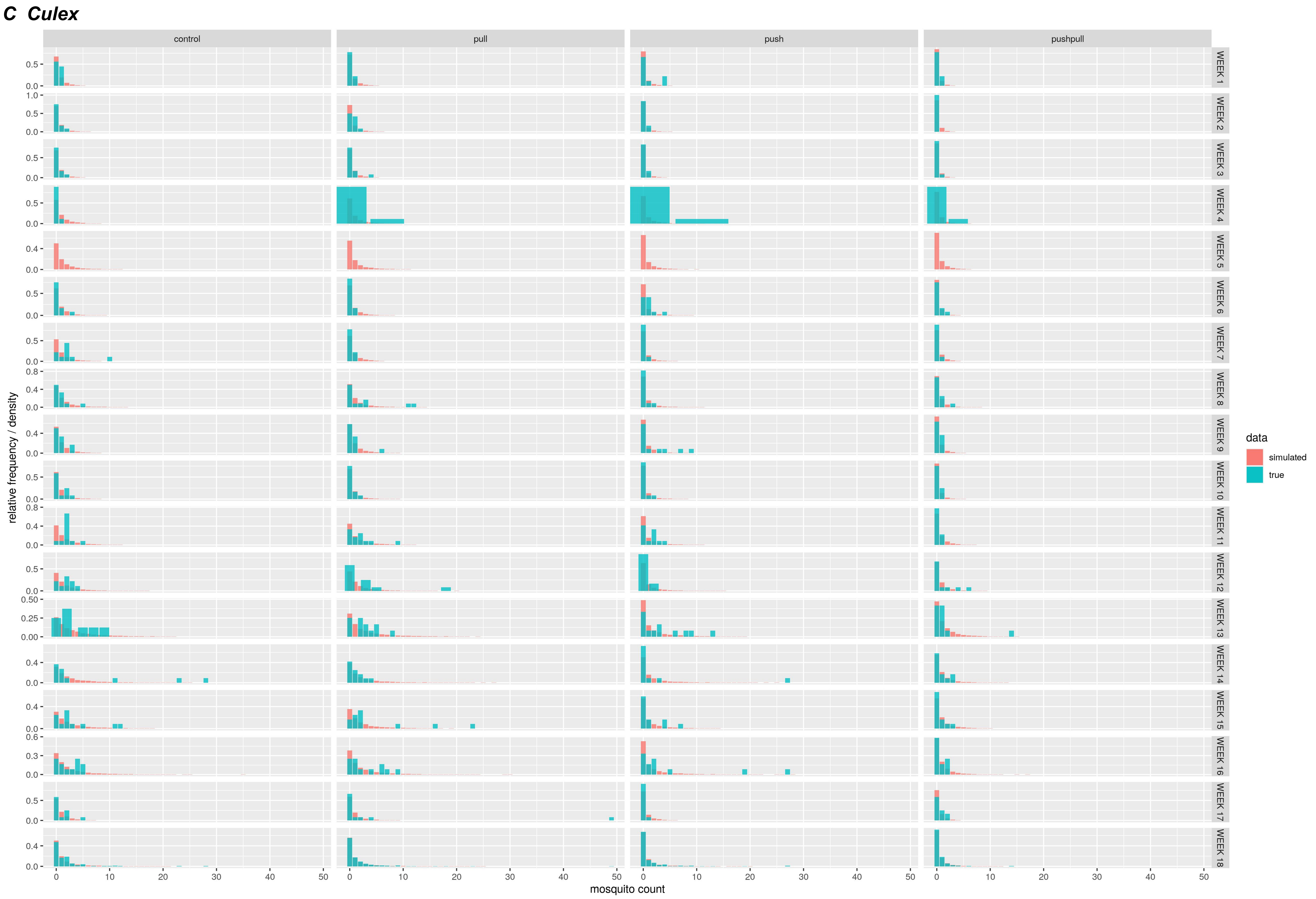

C Culex
control
pull
push
pushpull
WEEK 1
WEEK 2
WEEK 3
WEEK 4
WEEK 5
WEEK 6
WEEK 7
WEEK 8
WEEK 9
WEEK 10
WEEK 11
WEEK 12
WEEK 13
WEEK 14
WEEK 15
WEEK 16
WEEK 17
WEEK 18
relative frequency / density
mosquito count
data
simulated
true
