# Supplementary material for: A randomized, double-blind placebo-control study assessing the protective efficacy of an odour-based ‘push–pull’ malaria vector control strategy in reducing human-vector contact
Source: Sci Rep. 2023 Jul 11;13:11197. doi: 10.1038/s41598-023-38463-5 (PMC10336143; doi:10.1038/s41598-023-38463-5)

Supplementary Figure S5

to **"A randomized, double-blind placebo-control study assessing the protective efficacy of an odour-based 'push-pull' malaria vector control strategy in reducing human-vector contact"** by Ulrike Fillinger, Adrian Denz, Margaret M. Njoroge, Mohamed M. Tambwe, Willem Takken, Joop J.A. van Loon, Sarah J. Moore, Adam Saddler, Nakul Chitnis, Alexandra Hiscox

Per house estimates (posterior probability distributions) of the mean bite counts outdoors (A) and indoors (B), under the different interventions. 'HOUSE 13' denotes a further, unknown house and thus corresponds to the estimates for an arbitrary house as shown with red curves in Figures 1 and 2 of the article. The estimates shown here include the predicted variability with respect to the weeks. Note that the estimates are averaged over two models with the intervention location parameter depending either on the house or the week, while the baseline (control) location parameter depended on both house and week simultaneously for both models.

A outdoor

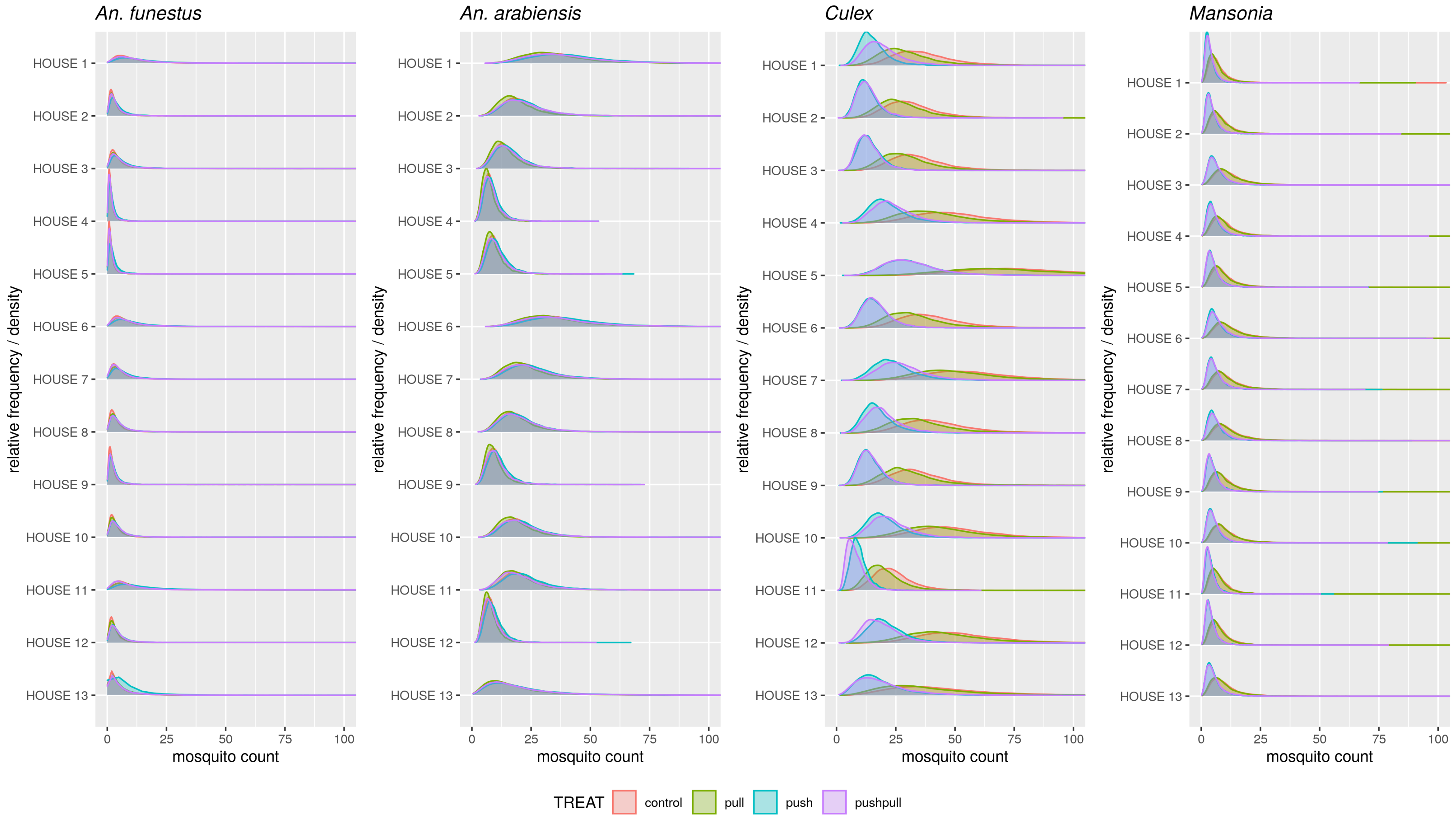

B indoors

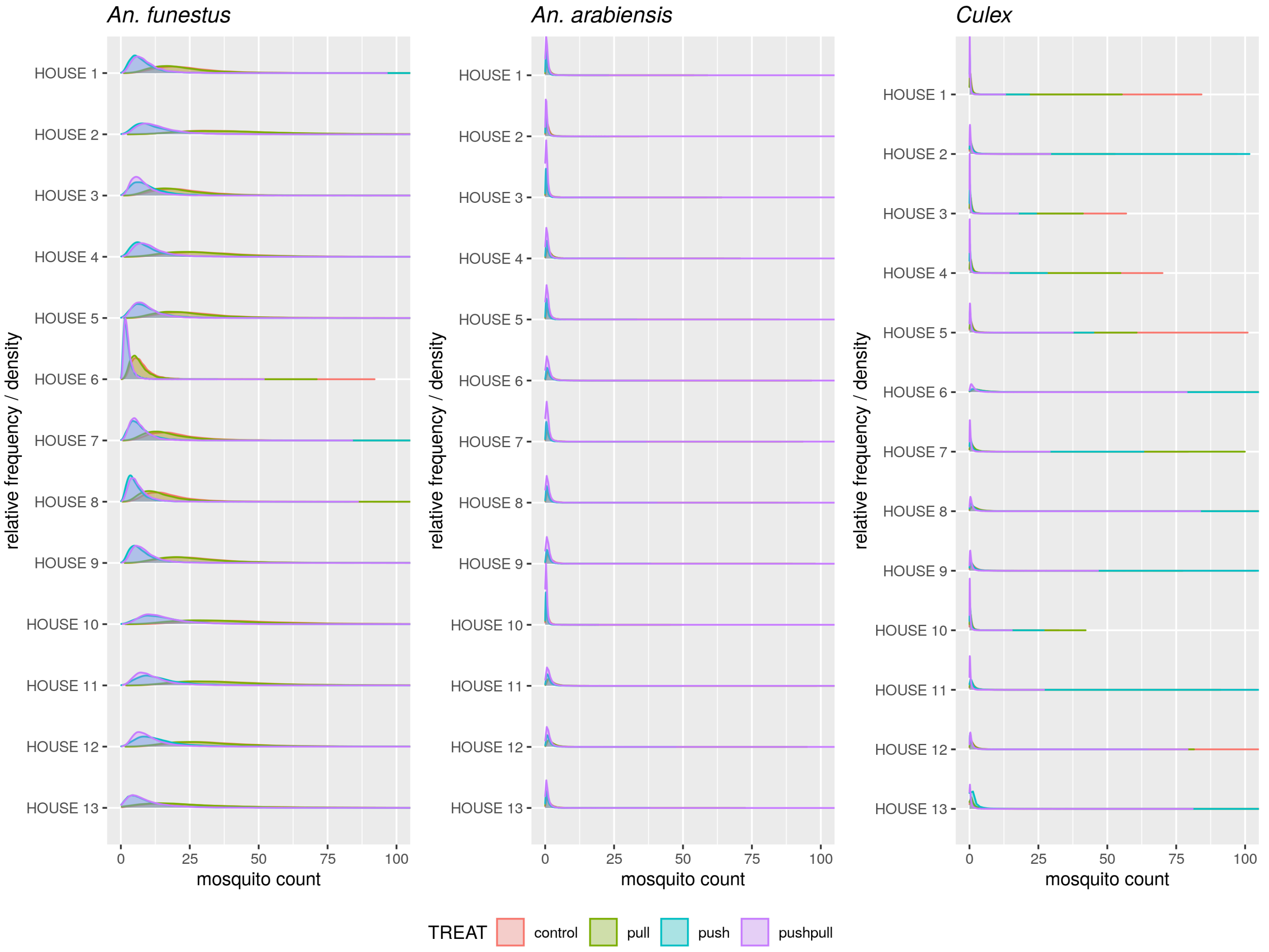

Supplement: Supplementary file 5 — Supplementary Figure S5. [file 41598_2023_38463_MOESM5_ESM.pdf]
