# Supplementary material for: A randomized, double-blind placebo-control study assessing the protective efficacy of an odour-based ‘push–pull’ malaria vector control strategy in reducing human-vector contact
Source: Sci Rep. 2023 Jul 11;13:11197. doi: 10.1038/s41598-023-38463-5 (PMC10336143; doi:10.1038/s41598-023-38463-5)

Supplementary Figure S6

to **"A randomized, double-blind placebo-control study assessing the protective efficacy of an odour-based 'push-pull' malaria vector control strategy in reducing human-vector contact"** by Ulrike Fillinger, Adrian Denz, Margaret M. Njoroge, Mohamed M. Tambwe, Willem Takken, Joop J.A. van Loon, Sarah J. Moore, Adam Saddler, Nakul Chitnis, Alexandra Hiscox

Per week estimates (posterior probability distributions) of the mean bite counts outdoors (A) and indoors (B), under the different interventions. WEEK 18' denotes a further, unknown week and thus corresponds to the estimates for an arbitrary week as shown with red curves in Figures 1 and 2 of the article. In WEEK 5 no measurements were taken, hence the estimates shown are identical to WEEK 18. The estimates shown here include the predicted variability with respect to the houses. Note that the estimates are averaged over two models with the intervention location parameter depending either on the week or the house, while the baseline (control) location parameter depended on both week and house simultaneously for both models.

A outdoor

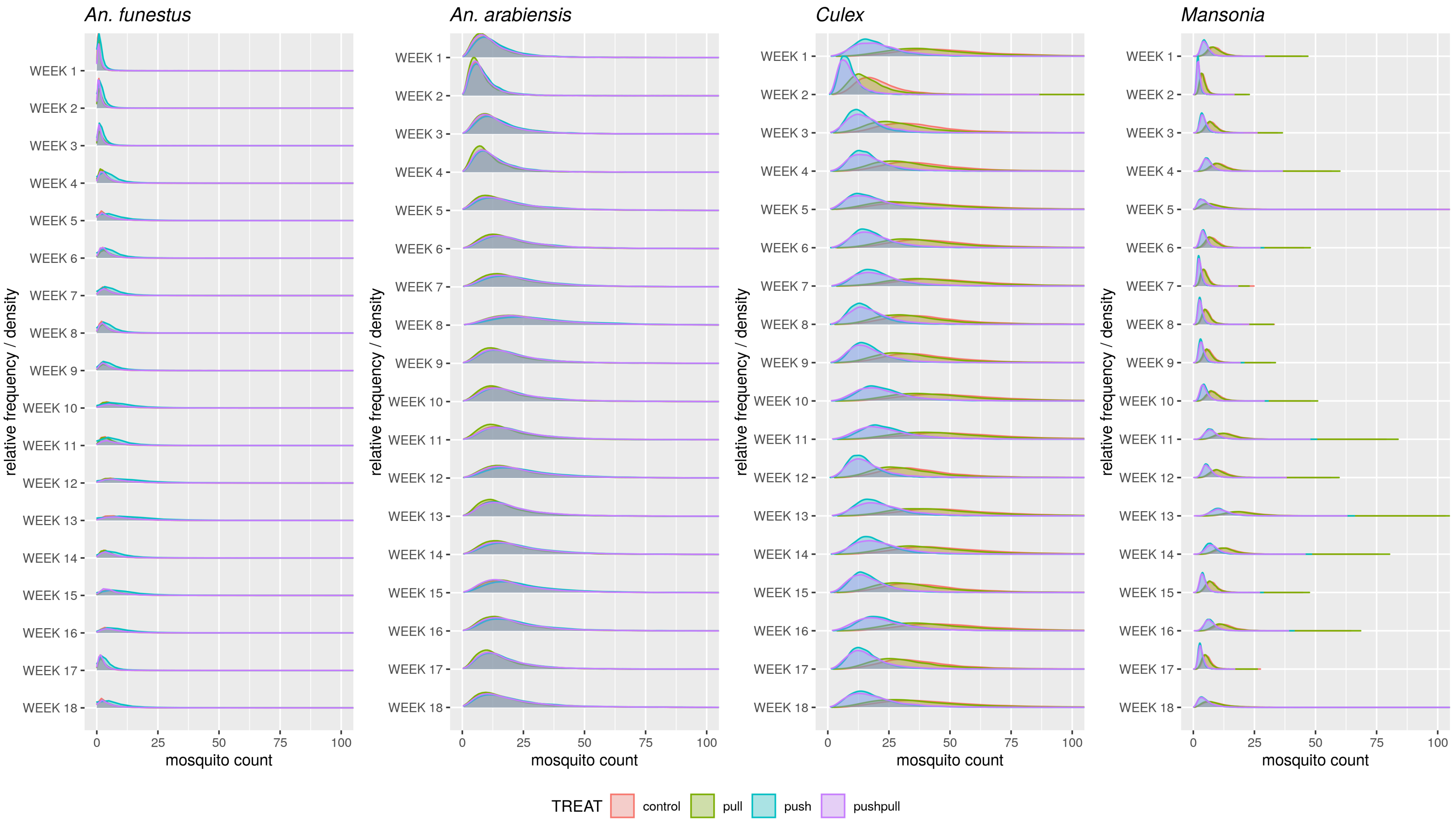

B indoors

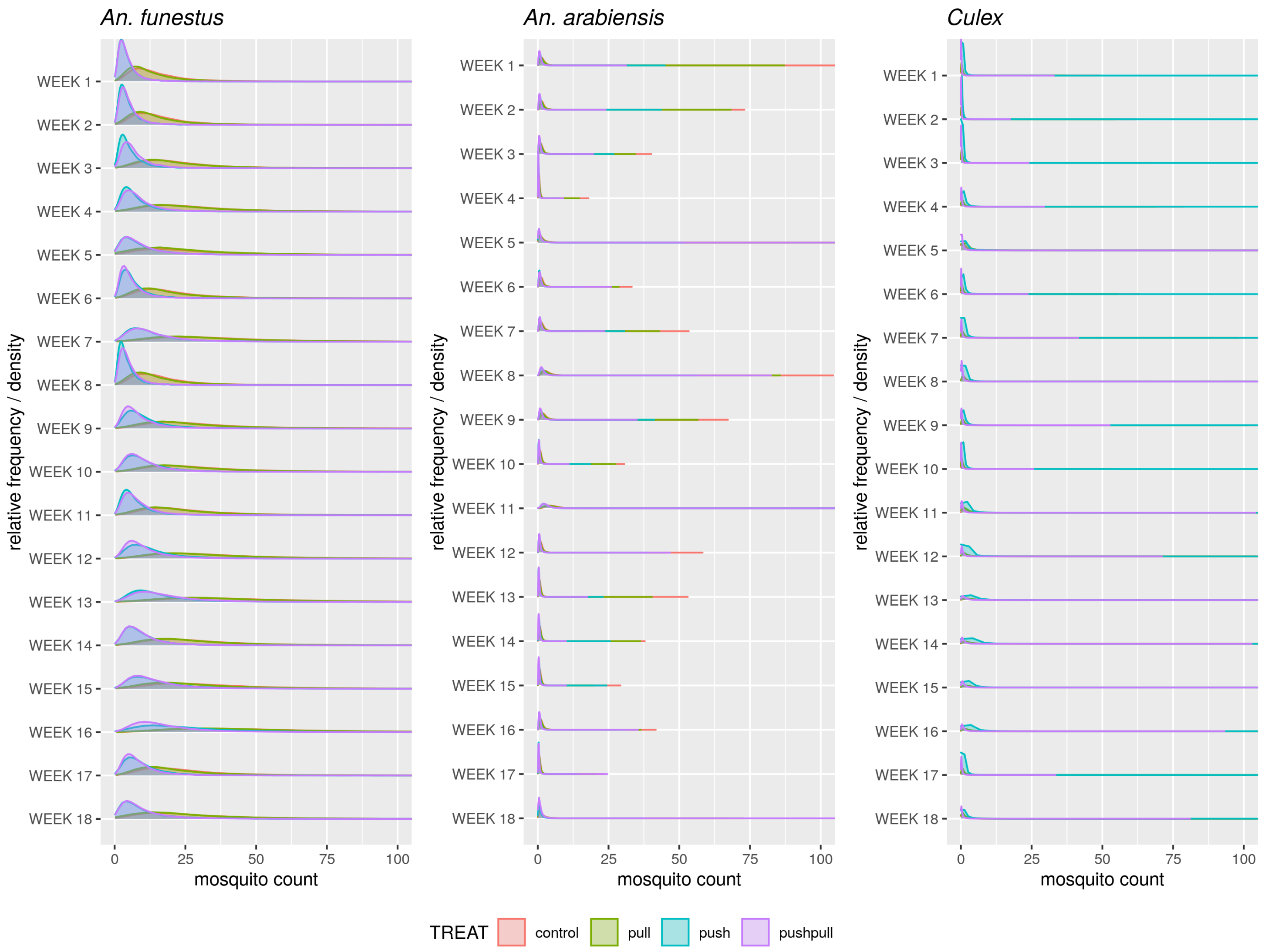

Supplement: Supplementary file 6 — Supplementary Figure S6. [file 41598_2023_38463_MOESM6_ESM.pdf]
