# Supplementary material for: A randomized, double-blind placebo-control study assessing the protective efficacy of an odour-based ‘push–pull’ malaria vector control strategy in reducing human-vector contact
Source: Sci Rep. 2023 Jul 11;13:11197. doi: 10.1038/s41598-023-38463-5 (PMC10336143; doi:10.1038/s41598-023-38463-5)
Supplement: Supplementary file 7 — Supplementary Information 1. [file 41598_2023_38463_MOESM7_ESM.pdf]

Supplementary Methods

to **"A randomized, double-blind placebo-control study assessing the protective efficacy of an odour-based 'push-pull' malaria vector control strategy in reducing human-vector contact"** by Ulrike Fillinger, Adrian Denz, Margaret M. Njoroge, Mohamed M. Tambwe, Willem Takken, Joop J.A. van Loon, Sarah J. Moore, Adam Saddler, Nakul Chitnis, Alexandra Hiscox

Detailed model exposition

Let y_i denote the ith data point of either the outdoor human landing or indoor light trap collections, with $1 \leq i \leq n$ and n denoting the number of data points. We group the data points into two layers of hierarchical groups: by the house at which the data point was collected (house-group) and by the week in which the data point was collected (week-group). We then allow a given parameter of the negative binomial model (first level model) to vary by group by introducing group-specific error terms (random effects) specified by the second-level model. For both house and week error terms, independent Gaussian second level models were chosen, so that the combined (house-week) group-specific error terms had a Gaussian distribution as well. Hence, the second-level model is equivalent to a Gaussian second-level model with grouping by all house-week combinations, with the advantage of being able to distinguish the variance induced by the house and the week.

We write the hierarchical (multi-level) model in terms of group-specific errors/deviations (see[1] for other, equivalent representations of multilevel models) as

$$y_i \sim \mathrm{NB}(\alpha + X_i M_\beta + \eta_{j[i]} + \zeta_{k[i]} + \xi_{l[i]}^{(x[i])}, X_i \Phi)$$

$$\eta_j \sim \mathcal{N}(0, \sigma_\eta^2)$$

$$\zeta_k \sim \mathcal{N}(0, \sigma_\zeta^2)$$

$$\xi_l^{(x)} \sim \mathcal{N}(0, \sigma_{\beta(x)}^2) \quad \text{for } x \in \{C, T, R, P\},$$

where

- NB denotes the negative binomial distribution parameterised by the log of its mean (called 'log-rate' here) and a dispersion parameter (being the inverse of (variance – mean) / mean^2),
- α denotes the mean log-rate for the controls (mean over all hierarchical groups),
- X_i denotes a column vector of length 4 with entries in $\{0, 1\}$ encoding the intervention deployed at y_i,
- $M_\beta = (0, \mu_{\beta(T)}, \mu_{\beta(R)}, \mu_{\beta(P)})$ denotes the vector of mean intervention effect

parameters (mean over all hierarchical groups),

- C denotes the placebo intervention (control),
- T denotes the trap intervention,
- R denotes the repellent intervention,
- P denotes the push-pull intervention,
- $\eta_{j[i]}$ denotes the deviation of the log-rate parameter for the house-group $j[i]$ from the mean log-rate, where $j[i]$ denotes the house-group to which y_i belongs,
- $\zeta_{k[i]}$ denotes the deviation of the log-rate parameter for the week-group $k[i]$ from the mean log-rate, where $k[i]$ denotes the week-group to which y_i belong,
- $\xi_{l[i]}^{(x[i])}$ denotes the deviation of the intervention effect parameter for intervention $x[i]$ for the hierarchical group $l[i]$ from the mean intervention effect parameter for intervention $x[i]$, where $l[i]$ either denotes the house-group or the week-group to which y_i belongs (i.e. either $l = j$ or $l = k$),
- $x[i]$ denotes the intervention that was deployed at y_i,
- $\Phi = (\phi_C, \phi_T, \phi_R, \phi_P)$ denotes the vector of dispersion parameters corresponding to the different interventions,
- σ_η denotes the standard deviation of the house-specific log-rates from the mean log-rate,
- σ_ζ denotes the standard deviation of the week-specific log-rates from the mean log-rate, and
- $\sigma_{\beta^{(x)}}$ denotes the standard deviation of the either house- or week-specific intervention effect parameter from the mean intervention effect parameter for intervention (x) (note that $\sigma_{\beta^{(C)}} = 0$).

Model variants and comparison

In order to investigate the impact of the experimental design, additional models with different hierarchical structures were fitted and their goodness of fit was compared. Also, a model with a different functional relationship of the intervention effect, a model that couples the deviation of the indoor and outdoor means by house and week, and a model with a hierarchical structure for the dispersion parameter instead of the location parameter were explored. Additionally, models with a step-like increase in mosquito density over time (either on a week or day scale) were fitted to investigate the impact of the rainy season which started about 4 weeks into the trial. All tested model variants are displayed in the Table below. The two models that were averaged for the final inference are denoted ‘5_1’ and ‘9_1’ here.

For all model variants the expected log posterior density (elpd) with respect to both the *An. funestus sl* and *An. arabiensis* data was estimated by leave-one-out cross-validation (LOO-CV) with pareto-smoothed importance sampling[2] by use of the R package loo[3]. The pareto-k values were investigated as a means of diagnosing misspecification. For the two models used for the final inference, at most 10 data points out of about 700 had pareto-k values higher than 0.5 (all lower than 1). For data points with pareto-k value higher than 0.5[2], exact LOO-CV was performed. The table below shows the estimated elpd's for all models fitted to the *An. arabiensis* outdoor densities. We consider two models with elpd_diff greater than 4 and se_elpd_diff smaller than elpd_diff as distinguishable in terms of their predictive accuracy. The two models that were averaged for the final inference ('5_1' and '9_1') performed best, together with model '4_4', which contrasts by not accounting for the variability of the protective efficacy by house or week. This indicates that the protective efficacy was relatively uniform across both houses and weeks of the trial. he two models that were averaged for the final inference ('5_1' and '9_1') were among the best fitting models for all mosquito densities (elpd estimates not shown).

Note that the Bayes factor between two models could be estimated by LOO-CV and the difference of the elpd's of two models could therefore be interpreted as the log of their Bayes factor (assuming a uniform prior on the two models). This would assume that one of the two compared models is correct (M-closed case[4]), and corresponds to a hypothesis testing situation. However, the aim of this model comparison exercise was to show that the models that were used for the final inference -- chosen based on their structure accounting for the dependencies between data points by experimental design – actually perform very well in comparison to alternative models.

Supplementary Table: model variants and their goodness of fit with respect to *An. arabiensis* outdoor density data as measured by human landing catches (HLC)

model name	Hierarchical grouping for baseline mean	Hierarchical grouping for intervention parameter	Other differences with respect to chosen models (5_1 and 9_1)	elpd, se_elpd (for *An. arabiensis* outdoors)
2_3	-	-	-	-3707.2, 45.5
3_3	house	-	-	-3539.3, 45.2
4_1	house	-	logistic function of the week for baseline (control) mean	-3496.4, 47.0
4_1_day	house	-	logistic function of the day for baseline (control) mean	-3537.7, 45.4
4_4	house + week	-	-	**-3411.4, 43.2**
4_4_mult1	house + week	-	multiplicative effect on log-count scale	-3903.4, 64.2
4_4_mult2	house + week	-	multiplicative effect on count scale	-3437.6, 40.9
4_4_singletheta	house + week	-	binding house effect on baseline log-rate for indoor and outdoor	-3426.1, 43.3
4_4_singletau	house + week	-	binding week effect on baseline log-rate for indoor and outdoor	-3458.0, 46.0
4_4_psiweek	house + week	-	week grouping on dispersion parameter instead of mean parameter	-3475.2, 41.5
5_1	house + week	house	-	**-3416.0, 43.2**
6_1	week	-	-	-3620.5, 41.7
7_1	house	house	-	-3535.0, 44.4
8_1	week	week	-	-3584.9, 42.1
9_1	house + week	week	-	**-3414.3, 43**

The three best fitting models are highlighted in green. Models 5_1 and 9_1 were averaged for the presented results.

References

1. Gelman, A. & Hill, J. *Data analysis using regression and multilevel/hierarchical models*. (Cambridge University Press, 2007).
2. Vehtari, A., Gelman, A. & Gabry, J. Practical Bayesian model evaluation using leave-one-out cross-validation and WAIC. *Stat Comput* **27**, 1413–1432 (2017).
3. Vehtari, A. *et al.* loo: Efficient leave-one-out cross-validation and WAIC for Bayesian models. *https://mc-stan.org/loo/* (2020).
4. Yao, Y., Vehtari, A., Simpson, D. & Gelman, A. Using Stacking to Average Bayesian Predictive Distributions (with Discussion). *Bayesian Anal.* **13**, 917–1007 (2018).
