# Supplementary material for: A randomized, double-blind placebo-control study assessing the protective efficacy of an odour-based ‘push–pull’ malaria vector control strategy in reducing human-vector contact
Source: Sci Rep. 2023 Jul 11;13:11197. doi: 10.1038/s41598-023-38463-5 (PMC10336143; doi:10.1038/s41598-023-38463-5)
Supplement: Supplementary file 8 — Supplementary Information 2. [file 41598_2023_38463_MOESM8_ESM.pdf]

Supplementary Note

to **“A randomized, double-blind placebo-control study assessing the protective efficacy of an odour-based ‘push-pull’ malaria vector control strategy in reducing human-vector contact”** by Ulrike Fillinger, Adrian Denz, Margaret M. Njoroge, Mohamed M. Tambwe, Willem Takken, Joop J.A. van Loon, Sarah J. Moore, Adam Saddler, Nakul Chitnis, Alexandra Hiscox

None of the interventions increased the dispersion of the mosquito counts

The scale parameter of the negative binomial distribution is defined as the square of the mean divided by the difference between the variance and the mean, and controls the overdispersion of the negative binomial distribution as compared to a Poisson distribution. The smaller the scale parameter the stronger is the overdispersion, while a scale parameter tending to infinity recovers the Poisson case. In our model, the scale parameter was allowed to vary by intervention, and the corresponding estimates confirmed that none of the interventions increased the dispersion of the mosquito counts. This is important, as an increase of the dispersion due to an intervention would indicate an unwanted increase of heterogeneity in malaria transmission under the intervention. In fact, to the contrary, the spatial repellent decreased the dispersion of the outdoor counts of both *An. funestus* and *An. arabiensis* as compared to the control, and the trap decreased the dispersion of the outdoor counts of both *An. arabiensis* as compared to the control. However, in none of these cases the intervention had an effect on the mean mosquito count.

The scale parameter of the negative-binomial distribution was much less variable across different weeks than the location parameter (mean), as shown by a less well fitting alternative model that allowed for variation of the scale parameter by week and assumed constant means across weeks (see Supplementary Methods). This indicates that the total number of mosquitoes varied strongly across weeks, but the variability of the daily counts for a given week did not vary much across weeks.

Table: Scale parameter of the negative binomial distribution representing outdoor and indoor mosquito densities as measured by human landing catches (HLC) and light traps, respectively, under the different interventions.

	Placebo control	Pull (Odour-baited Suna trap)	Push (Transfluthrin-treated fabric strip on roof eave gaps)	Push-pull (Combination of transfluthrin strip and odour-baited traps)
**OUTDOOR Human landing collections**				
Anopheles funestus	1.4, [0.9,1.9]	1.5, [1,2]	**3.2, [1.9,4.6]**	2.9, [1.7,4.3]
Anopheles arabiensis	2.5, [1.8,3.1]	**4.3, [3.1,5.6]**	**5.2, [3.7,6.7]**	3.5, [2.6,4.6]
Culex	2.1, [1.6,2.5]	2.1, [1.6,2.6]	2, [1.5,2.5]	1.7, [1.3,2.2]
Mansonia	1.1, [0.8,1.4]	1.7, [1.2,2.3]	1.5, [1,2.1]	1.5, [1,2]
**INDOOR light trap collections**				
Anopheles funestus	1.2, [0.9,1.5]	1.6, [1.3,2]	1.3, [1,1.7]	1.7, [1.2,2.3]
Anopheles arabiensis	0.8, [0.5,1.1]	1.1, [0.7,1.7]	0.9, [0.5,1.5]	1.2, [0.5,2.1]
Culex	1, [0.6,1.6]	0.8, [0.5,1.2]	0.5, [0.3,0.7]	1.3, [0.3,2.6]

Estimates of the scale parameter are expressed as the mean and 95% highest density credible interval (HDI) of the posterior distribution. A change of the scale parameter with respect to the control of at least 95% credibility is highlighted in green.
