# Supplementary material for: A randomized, double-blind placebo-control study assessing the protective efficacy of an odour-based ‘push–pull’ malaria vector control strategy in reducing human-vector contact
Source: Sci Rep. 2023 Jul 11;13:11197. doi: 10.1038/s41598-023-38463-5 (PMC10336143; doi:10.1038/s41598-023-38463-5)
Supplement: Supplementary file 9 — Supplementary Tables. [file 41598_2023_38463_MOESM9_ESM.pdf]

Supplementary Table

to **"A randomized, double-blind placebo-control study assessing the protective efficacy of an odour-based 'push-pull' malaria vector control strategy in reducing human-vector contact"** by Ulrike Fillinger, Adrian Denz, Margaret M. Njoroge, Mohamed M. Tambwe, Willem Takken, Joop J.A. van Loon, Sarah J. Moore, Adam Saddler, Nakul Chitnis, Alexandra Hiscox

Supplementary Table S1: Mean outdoor and indoor mosquito densities as measured by human landing catches (HLC) and light traps, respectively, and corresponding relative changes due to the interventions, with interval estimates for an arbitrary house-week.

	Placebo control	Pull (Odour-baited Suna trap)	Push (Transfluthrin-treated fabric strip on roof eave gaps)	Push-pull (Combination of transfluthrin strip and odour-baited traps)
**OUTDOOR Human landing collections**				
Anopheles funestus	6.1 (0.4, 29.6)	7.4 (0.4, 36.9)	9.1 (0.5, 43.7)	7.4 (0.4, 37.9)
		22.4% (-39.9%, +122.3%)	48.4% (-22.8%, +162.8%)	22.8% (-50.8%, +157.2%)
Anopheles arabiensis	22.8 (3.7 , 78.6)	20.3 (3.2, 70.8)	23.9 (3.8, 82.7)	22.6 (3.4, 80)
		-10.5% (-34.4%, +18.3%)	5.4% (-19.8%, +36.6%)	-0.7% (-35.9%, +48.8%)
Culex	46.3 (13.8 , 113.2)	40 (10.8, 104.3)	19.3 (5.2, 50)	20.7 (4.7, 58.2)
		-13.2% (-46.6%, +30.6%)	**-58.3% (-74.5%, -36.6%)**	**-55.2% (-80.6%, -12%)**
Mansonia	10.4 (2.5 , 28.5)	9.8 (2.3, 27.8)	5.5 (1.2, 15.5)	5.7 (1.2, 16.6)
		-5.3% (-35.4%, +32.5%)	**-47% (-65.8%, -19.9%)**	**-45% (-68.9%, -8.8%)**
**INDOOR light trap collections**				
Anopheles funestus	33.3 (4.7 , 117.4)	31.2 (3.9, 112.5)	11.8 (1.2, 47.4)	11.2 (1.3, 43.1)
		-6.3% (-49.4%, +62.3%)	**-64.1% (-88.9%, -11.5%)**	**-66.5% (-85.5%, -34.2%)**
Anopheles arabiensis	2 (0.2 , 7.4)	1.8 (0.2, 7.4)	1.1 (0.1, 4.6)	1.1 (0.1, 4.4)
		-6.3% (-61.8%, +85.2%)	-41.2% (-76.9%, +21.2%)	-44.3% (-78.5%, +18.6%)
Culex	2.1 (0.1 , 10)	2.1 (0.1, 11.3)	1.8 (0, 9.6)	0.8 (0, 4.1)
		+1.2% (-73.6%, +160.5%)	-11.8% (-89.4%, +195.7%)	-61.3% (-92.1%, +5.5%)

This table reports the findings on reduction in host-seeking mosquito density (primary outcome) with uncertainty quantification under the arbitrary house-week view. In contrast, in Table 1 of the main text the uncertainty was quantified with respect to an average house and average week. All results are expressed as the mean and 95% highest density credible interval (HDI) of the posterior distribution of the outcome, with interval estimates (HDIs) corresponding to an arbitrary house-week. A relative change has 95% credibility if its HDI excludes 0% (unity). A positive protective efficacy (negative relative change) with 95% credibility is highlighted in green. Note that the means of the posterior distribution of both, the mosquito density and the relative change, are identical to the corresponding means under the average house-week view as reported in Table 1 of the main text, the small discrepancies in the means are solely due to finite sample calculations.
